# Localization and Composition of Fructans in Stem and Rhizome of *Agave tequilana* Weber var. azul

**DOI:** 10.3389/fpls.2020.608850

**Published:** 2021-01-20

**Authors:** Arely V. Pérez-López, June Simpson, Malcolm R. Clench, Alan D. Gomez-Vargas, José J. Ordaz-Ortiz

**Affiliations:** ^1^Department of Genetic Engineering, CINVESTAV Unidad Irapuato, Irapuato, Mexico; ^2^Centre for Mass Spectrometry Imaging, Biomolecular Sciences Research Centre, Sheffield Hallam University, Sheffield, United Kingdom; ^3^Metabolomics and Mass Spectrometry Laboratory, National Laboratory of Genomics for Biodiversity, Unidad de Genómica Avanzada (CINVESTAV), Irapuato, Mexico

**Keywords:** fructan, *Agave tequilana*, mass spectrometry imaging, ion mobility separation, degree of polymerization, fructan isoform, fructan metabolism, collision cross section

## Abstract

Methodology combining mass spectrometry imaging (MSI) with ion mobility separation (IMS) has emerged as a biological imaging technique due to its versatility, sensitivity and label-free approach. This technique has been shown to separate isomeric compounds such as lipids, amino acids, carboxylic acids and carbohydrates. This report describes mass spectrometry imaging in combination with traveling-wave ion mobility separation and matrix-assisted laser desorption/ionization (MALDI). Positive ionization mode was used to locate fructans on tissue printed sections of *Agave* rhizome and stem tissue and distinguished fructan isoforms. Here we show the location of fructans ranging from DP3 to DP17 to be differentially abundant across the stem tissue and for the first time, experimental collision cross sections of endogenous fructan structures have been collected, revealing at least two isoforms for fructans of DP4, DP5, DP6, DP7, DP8, DP10, and DP11. This demonstrates that complex fructans such as agavins can be located and their isoforms resolved using a combination of MALDI, IMS, and MSI, without the need for extraction or derivatization. Use of this methodology uncovered patterns of fructan localization consistent with functional differences where higher DP fructans are found toward the central section of the stem supporting a role in long term carbohydrate storage whereas lower DP fructans are concentrated in the highly vascularized central core of rhizomes supporting a role in mobilization of carbohydrates from the mother plant to developing offsets. Tissue specific patterns of expression of genes encoding enzymes involved in fructan metabolism are consistent with fructan structures and localization.

## Introduction

Fructans are branched or linear polyfructose molecules that accumulate in 15% of angiosperms (Hendry, 1993) in families of both dicotyledons and monocotyledons (Van den Ende, 2013; Versluys et al., 2018; Pérez-López and Simpson, 2020). They can be defined as compounds where one or more fructosyl-fructose linkage constitutes the majority of linkages and they may or may not contain a D-glucosyl (Lewis, 1993). The glycosidic linkages of fructans can be either β(2→1), β(2→6) or both in order to form polymers, with a degree of polymerization (DP) equal or greater than ten, or oligomers with a DP range between 3 and 9 (Peukert et al., 2014).

One of the most important fructan producing families is the Asparagaceae, which includes asparagus, onion and agave species. The latter are undoubtedly part of the Mexican landscape and *Agave tequilana* Weber var. azul is an important crop in Mexican agriculture. In these Crassulacean Acid Metabolism (CAM) plants fructans are the main storage carbohydrates, providing the raw material for tequila and with potential to be exploited as a renewable bioenergy source (Davis et al., 2011, 2019; Escamilla-Treviño, 2012). Agave fructans are also exploited as dietary supplements due to their benefits as prebiotics with wide ranging applications for human health (Gomez et al., 2010; Márquez-Aguirre et al., 2016).

Agave fructans were first reported to be inulin types and described as the principal storage carbohydrates in *A. tequilana* and *A. americana* (Sanchez-Marroquin and Hope, 1953; Bhatia and Nandra, 1979). More recently (Lopez et al., 2003) demonstrated that fructans from *A. tequilana* Weber var. azul are a complex mixture of fructo-oligosaccharides containing mainly β(2→1) and β(2→6) linkages, with internal neoseries fructans and external graminan type fructans. To date, these complex and highly branched fructans have only been found and described in agaves and closely related species and they are commonly referred to as agavins. The presence of complex fructan polymers in *A. tequilana* suggests that up to four different fructosyltransferase activities are involved in their biosynthesis and fructan exo-hydrolases specific for different terminal bonds are responsible for fructan degradation (Cimini et al., 2015; Versluys et al., 2018; Morales-Hernández et al., 2019; Tarkowski et al., 2019; Pérez-López and Simpson, 2020).

In terms of biological function, fructans constitute the main form of carbohydrate storage in *Agave* species, with starch playing a secondary and temporary role in specific tissues (Zavala-García et al., 2018). In contrast, fructans are found in all agave tissues including roots, rhizomes and flowers although they accumulate to the highest levels in the oversized stem. More recently, based on specific properties, the importance of fructan metabolism in many adaptive traits of *Agave* species is beginning to be recognized (Avila de Dios et al., 2015; Pérez-López and Simpson, 2020). Fructans are highly soluble (Morales-Hernández et al., 2019) and strongly associated with succulence, can be transported through the phloem and can associate with plant membranes. These properties have led to the hypotheses that fructans participate in water storage mechanisms, can provide protection to membranes during desiccation due to drought or freezing, may act as signaling molecules such as microbe-associated molecular patterns (MAMP) and/or damage-associated molecular patterns (DAMP) responses and could also be involved in osmoregulation during flowering (Vijn and Smeekens, 1999; Van den Ende, 2013; Versluys et al., 2017). A deeper understanding of the roles of fructans in adaptation of *Agave* species to harsh desert environments is essential for the sustainable exploitation of a wider range of agave germplasm and this knowledge could potentially be used to support the development of drought tolerance in more traditional crops.

In order to understand the putative functional roles of agave fructans it is essential to determine the distribution, detailed tissue localization and putative mobility of fructans of different sizes and complexities and how their spatial/temporal distribution relates to the regulation of fructan metabolism at the molecular level. Previously, detailed structural analysis of agave fructans has been carried out using high performance anion-exchange chromatography with pulsed amperometric detection (HPAEC-PAD), size exclusion chromatography (SEC), reversed phase high performance liquid chromatography (rpHPLC) with the use of reductive methylation, as well as nuclear magnetic resonance (NMR). By using these methodologies, it was shown that the degree of polymerization (DP) of fructans from agave plants increases as plants get older, for example, molar mass analysis of water-extracted fructans from the stem of a 6 years old *A. tequilana* plant showed broad DP distributions ranging from 3 to 70 with a mean value of 30 for samples from the center of the stem and the intersection connecting stem and leaves (Praznik et al., 2013). Interestingly HPAEC-PAD fructan profiles of *A. tequilana* at different ages have shown several isomers in relation to oligomers between DP3 to DP12 (Mellado-Mojica and López, 2012), demonstrating the complexity of agavins when compared with other fructan producing plant species such as *C. intybus*, *A. cepa* and *A. sativum* (Mellado-Mojica and López, 2012).

The most frequently used technique for separation and structural determination of agave fructans has been HPAEC-PAD, with the disadvantage that only oligomers of DP less than 15 can be resolved and long run times are required. More recently separation and quantification of fructan oligomers in crude ryegrass extracts ranging from DP3 to DP49 have been carried out successfully with the use of porous graphitized carbon chromatography coupled to negative ion electrospray ionization mass spectrometry (ESI-MS) (Robinson et al., 2007; Antonio et al., 2008; Harrison et al., 2011). Furthermore, the analysis of fructans up to DP100 has been achieved using high-resolution mass spectrometry (HRMS) with the aid of Orbitrap technology that successfully distinguished and monitored multiple charged ions (Harrison et al., 2011). In general, these methods are invasive, involving extraction and processing of samples where the underlying tissue specific localization is lost.

*In situ* determination of the presence of fructans, however, is difficult since no specific histological methods to distinguish fructans from other carbohydrates are available. Although fructan crystals have been identified in *Campuloclinium chlorolepis* roots using PAS staining (Vilhalva et al., 2011), and (Mellado-Mojica et al., 2016) have shown that fructans are also present in isolated vacuoles of agave stems, these methods are cumbersome or not feasible over a range of tissues or species. An option to address this problem is the use of matrix-assisted laser desorption/ionization-mass spectrometry imaging (MALDI-MSI) which has emerged as a biological imaging technique within the last 20 years because of its versatility, sensitivity, and label-free approach. MSI has been applied to visualize small molecules such as flavonoids (Cha et al., 2008), saponins (Li et al., 2014), lipids (Dueñas et al., 2017), and glucosides (Schmidt et al., 2018) in plant tissues and has been also applied to visualize water soluble oligosaccharides in wheat stems (Robinson et al., 2007), fructans in developing barley grains and asparagus roots (Peukert et al., 2014; Witzel and Matros, 2020) and arabinoxylans and (1→3), (1→4) β-glucans in wheat and barley grains (Velickovic et al., 2016).

Although they present many advantages, the above techniques are unable to unambiguously resolve distinct fructan isomers. The combination of ion mobility spectrometry (IMS) and mass spectrometry imaging (MSI) represents a promising approach to overcome these limitations. IMS separates ions based on the time required to traverse a region of inert neutral gas under the influence of a weak electric field. Ion separation is based on size, shape and charge and used in conjunction with MS, drift times and *m/z* values can be determined (Struwe et al., 2016). Drift times can then be used to calculate an instrument-independent, rotationally averaged collision cross-section (CCS), which is an intrinsic value reflecting the structure of the ion and is unique for each molecule (Hofmann et al., 2015; Struwe et al., 2016). Theoretical CCS values can be determined for a large number of candidate ion structures which are generated by molecular modeling and the agreement between theoretical CCS values of the candidate structures and experimental CCS serves as an important criterion for selecting plausible structures (Lapthorn et al., 2015; Lee et al., 2017).

In previous reports, MALDI-MSI was carried out directly on thin sections of plant tissue, however, these samples have a short life span, involve histological expertise and cannot be applied to larger organs or tissue samples such as agave stem tissue. We addressed these drawbacks, by implementing a method of tissue printing on nylon membranes for analysis by MALDI-IMS-MSI of *Agave tequilana* stem and rhizome samples, thereby accommodating samples in a wide range of sizes, retaining the spatial organization of the fructan molecules within the tissue under analysis and facilitating storage and transportation of samples between laboratories. To our knowledge this is the first time that agave fructan ion mobility separation-mass spectrometry imaging (IMS-MSI) data allowing the determination of conformational information has been carried out. Mass spectrometry analyses of dissected and extracted tissue sections were also included for comparison. We provide detailed locations and patterns of fructan distribution with ion mobility data as a first attempt to discriminate the spatial resolution of isomeric fructans in central stem and rhizome tissues. Biochemical data was also compared with qRT-PCR analysis of genes encoding enzymes involved in fructan metabolism in *A. tequilana* and shown to broadly correlate with fructan distribution and putative function.

## Materials and Methods

### Biological Material

Three-year-old *Agave tequilana* Weber var. azul plants grown from bulbils and acclimatized in a greenhouse were used to carry out all experimental analysis. Plants were watered once per week and grown in pots of 3.5 L with a mixture of red volcanic rock and leaf litter (50:50).

### Extraction of Soluble Carbohydrates

Soluble carbohydrates were obtained from agave tissues by aqueous extraction from dry or fresh tissue. Stems of 3 individual 3 years old plants were dissected as shown in Supplementary Figure 1 and Figure 3A. For dry tissue extraction 0.5 g of sample were lyophilized (Freezone 4.5, LABCONCO), and ground to powder with a mortar. Similarly, for aqueous extraction from fresh tissue, 3 g of fresh dissected tissue of 4 plants was ground in a ball mill (TissueLyser II, QIAGEN). In both cases, 15 mL of distilled water were added to the sample and incubated at 75 °C for 30 min in a water bath. Samples were then centrifuged at 3,500 rpm at room temperature (RT) for 10 min (Allegra X-12R, Centrifuge Beckman Coulter). The supernatant was recovered, and the pellet was resuspended in 10 mL of distilled water and then incubated at 75 °C for 15 min. This second extraction was centrifuged under the same conditions as above. Both extractions were pooled, filtered through grade 1 filter paper (Whatman^®^) and freeze-dried. Extracts were stored tightly sealed at RT (modified from Avila de Dios et al., 2015).

### Matrix Assisted Laser Desorption Ionization Mass Spectrometry (MALDI-MS)

Matrix α-cyano-4-hydroxycinnamic acid, poly-(D/L)-alanine, polyethylene glycol and phosphorus red for mass spectrometry analysis were obtained from Merck. MS grade methanol, acetonitrile and formic acid were obtained from Sigma. For fructan extract profiling, samples extracted from lyophilized tissue were re-dissolved in acetonitrile: water [70:30] at a concentration of 1 mg/mL, while matrix *α*-cyano-4-hydroxycinnamic acid (CHCA) was prepared at 10 mg/mL in the same solution. Then, 0.5 *μ*L of sample were immediately added to 0.5 *μ*L of matrix solution on the MALDI plate and co-crystallized by dry air. Four spots per extract from 3 individual plants were analyzed in positive ionization mode with a mass range of 100 to 3000 Da. In the APL QSTAR Pulsar1 instrument (Applied Biosystems). The laser was set with power level 5 *μ*J (50%) and pulse rate of 1000 Hz. In the MALDI SYNAPT G1 and MALDI SYNAPT G2 instruments (Waters Corporation, United Kingdom) the laser energy was 250–300 Hz, trap collision energy 4 V, and transfer collision energy 30 V. For the time of flight (ToF) mass calibration in W mode, polyethylene glycol (PEG) and Phosphorous red (PhosphoRed) were used. Kestotriose, nystose, ff-nystose (Wako Chemicals), levan and inulin (Merck) were also used as analytical standard samples. Data analysis was performed using the MassLynx package v.4.1 (Waters Corporation, United Kingdom). For MALDI-MS analysis we used two extracts from three individual plants each, sectioned as shown in Figure 3A. Tandem mass spectrometry was performed for fructan extracts, precursor ions from DP3 to DP18 were selected and fragmented.

### Mass Spectrometry Imaging (MSI)

Tissue printing on nylon membranes was carried out as follows, samples of fresh tissue were cut into pieces 3–5 cm thick slices with a razor blade. The exposed face of the tissue was placed on a piece of nylon membrane (Biodyne^®^ B, Thermo Fisher Scientific) and hand pressed onto the flat surface for 30 s (around 1 Pa per cm^2^ of pressure was applied). A piece of filter paper was placed below the membrane to buffer the print (modified from de Fátima Rosas-Cárdenas et al., 2015). For tissue preparation with matrix application, profiles in manual mode were directly executed under the same conditions as the profiling for extracts. Tissue prints on nylon membranes were mounted onto MALDI plates with a double-sided adhesive tape (Sellotape^®^). For spraying, an automatic sprayer SunCollect (SunChrom, Germany) was used with a flow of 3 μL/min of matrix solution or samples were manually sprayed using the analyte sprayer of an LCT Premier XE TOF (Waters, United Kingdom) under similar conditions using a concentration of five mg/mL of *α*-cyano-4-hydroxycinnamic acid (CHCA) matrix in acetonitrile:water [70:30]. Trifluoroacetic acid (TFA) 1% [v/v] was used to coat samples ten times at room temperature to achieve a final matrix coverage of 0.2 mg/cm^2^. For sublimation, 300 mg of matrix were placed in a sublimation chamber under vacuum at 180°C and 4°C for 8 min. For sublimation and deposition, respectively, the increase in weight was measured in order to reach the optimal matrix coverage.

MSI analysis in the MALDI SYNAPT G1 and SYNAPT G2 (Waters, United Kingdom) were performed under similar conditions. Initially an area of the printed tissue was selected and saved as an image. The plate was placed in the scanner for an image to be taken in MassLynx software v. 4.1 (Waters, United Kingdom), a project was created using HDI imaging software (Waters, United Kingdom) and the desired area was selected and converted into a pixel-coordinate file. Laser beam diameter on the sample was 175 μm to achieve a pixel resolution of 50–100 μm and laser repetition was 1000 Hz. This file was opened and run from MassLynx as the standardized MS protocol. Data analysis, normalization based on matrix peak distribution and relative abundance, and co-localization analysis were carried out in the HDI imaging program v. 2.1 (Waters Corporation). Analysis in the APL QSTAR pulsar1 mass spectrometer (Applied Biosystems, United States), were carried out with a laser set with power level of 5 μJ (50%) and pulse rate of 1000 Hz, using BioMAP 3.6.0.4 software (Novartis Institutes for Biomedical Research) for data processing. MALDI images of tissue printed stem sections of 3 individual *A. tequilana* plants were obtained for transverse sections and of 2 individual plants in longitudinal sections.

### Ion Mobility Spectrometry-Mass Spectrometry (IMS-MS)

For the MALDI SYNAPT G1 and SYNAPT G2 analyses, both instruments comprised a traveling-wave ion mobility device, operated with the following settings: laser energy 300–250 Hz, trap collision energy 4 V, and transfer collision energy 30 V. The ion mobility cell was activated and operated with Nitrogen as drift gas, and the mass spectrometer was operated in positive ionization mode under HDMS mode, with a MS resolution of 10,000 in a drift range from 1 to 200 bins, and 0.25 ppm of lock mass tolerance, IMS wave velocity of 300 m/s and wave height 8 V. Mass spectrometry data was analyzed in MassLynx and DriftScope 2.8 software (Waters Corporation). IMS-MS was carried out on tissue printed stem sections (2 transverse and 2 longitudinal) and on extracts from dissected stem tissue from 4 individual plants (as shown in Figure 3A).

### Calculation of Experimental Collisional Cross Section (CCS) Ω*_*e*_*

Poly-(D/L)-alanine at a concentration of 7 mg/mL (prepared in H_2_O/ACN 50:50, v/v) was used for calibration and spotted with CHCA matrix in a MALDI plate as mentioned above for MALDI-MS under positive ionization mode. Calibration was carried out with oligomers from *n* = 5 to *n* = 14, covering a mass range from 374 to 1013 Da and a CCS range from 181 Å^2^ to 306 Å^2^ (Supplementary Table 1). CCS values were calculated following the procedure previously described by Bush et al. (2012). The drift time for each oligomer was recovered in the DriftScope 2.8 software (Waters, United Kingdom) and compared with a CCS database (Bush et al., 2012). Drift time versus collisional cross section values was plotted and used as a reference for fructan CCS calculation. Calculation of Collisional Cross Section via computational methods was carried out after drawing 3D structures for all fructan DPs and isoforms using ChemDraw v 16.0 (CambridgeSoft). Isoform 3D models were built using ChemDraw 3D software (CambridgeSoft) and energy optimized using molecular mechanics lowest energy conformers by applying the MM2 force field algorithm (Schnur et al., 1991; Dudek and Ponder, 1995) using ChemDraw v 16.0. Theoretical CCS values (Ω_*th*_) were obtained using Waters Driftscope v. 2.8 that uses a rapid implementation of the projection approximation method. Comparisons were made using 6 extracts and 2 tissue prints from 2 individual plants.

### Carbohydrate Staining

Fresh tissue from three plants was washed and then cooled to 4°C to be sectioned using a microtome blade. Alternatively, samples were hand sectioned with a scalpel or a razor blade and used immediately for staining.

The Periodic Acid-Schiff (PAS) stain for total carbohydrates was used for *in situ* carbohydrate localization. Samples were covered with 500 μL of periodic acid solution (0.5% v/v) and incubated at RT for 5 min and then washed with distilled water for 1 min. Then, 500 μL of Schiff reagent was added to the washed slides and left at RT for 15 min. For starch localization, Lugol dye (500 μL) was added to sample slides and incubated for 15 min. After staining, slides were washed for 1 min before being visualized under bright field and phase contrast microscopy (OLYMPUS BX60 microscope and Leica E24HD stereoscope (Zavala-García et al., 2018).

### Fructan Quantification

Fructan extracts from dissected stem samples as shown in Figure 3A (25 mg/mL) or an analytical standard (10 mg/mL), were quantified twice by enzymatic degradation, using the Megazyme kit in a scaled protocol [1:5] according to the manufacturer’s instructions and 50 μL of each sample were analyzed in a 96-well microplate at 410 nm in a UV plate reader (Epoch, BioTek). Four individual plants were sampled and analyzed in duplicate.

### Thin Layer Chromatography (TLC)

Fructan extracts and analytical standards glucose, fructose, sucrose (Merck), 1-kestotriose, 6-nystose, and fructofuranosyl-nystose 1 μL at a concentration of 5–50 mg/mL in water, were run on a silica-gel TLC plate in a mobile phase of butanol: acetic acid: water [50:25:25 v/v/v] three times for distances of 2.5, 5.0, and 7.5 cm, respectively, and dried between runs. Plates were developed with a solution of aniline 2% [v/v], diphenylamine 2% [w/v], phosphoric acid 15% [v/v] in acetone at 75°C for 15 min (Boekel Oven) (modified from Avila de Dios et al., 2015). Pooled extracts from dissected tissue (Figure 3A) from 2 individual plants were spotted and run on duplicate TLC plates.

### Expression Analysis

**FIGURE 1:**
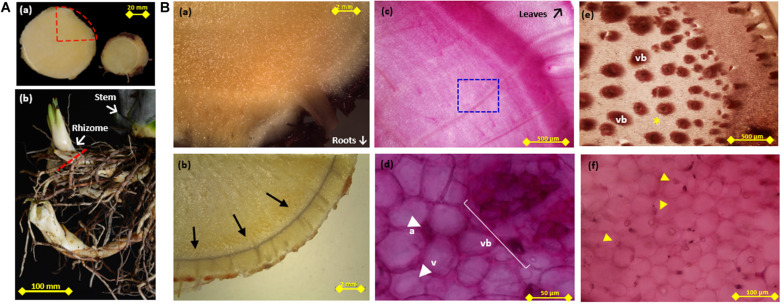
Periodic Acid-Schiff (PAS) and Lugol staining of agave stem and rhizome tissue. **(A)** (**a**-left) Cross section of agave stem tissue, dotted red triangle indicates the area shown in **(Ba–c)** (**a**-right). Transverse section of rhizome tissue from **(Be,f)** were obtained. **(Ab)** Example of rhizomes attached to stem tissue, dotted red line indicates a transverse section. **(Ba)** Unstained, **(b)** lugol stained (indicated by arrows), and **(c)** PAS stained triangular slices of transverse sections of stem tissue **(d)** Higher magnification of PAS stained stem tissue [blue square in **(c)**, showing vascular tissue (vb), vacuole (v), and apoplast (ap)]. **(e)** Periodic Acid-Schiff (PAS) stained triangular slices of transverse sections of rhizome indicating vascular tissue (vb), **(f)** Higher magnification of rhizome tissue corresponding to non-vascular tissue indicated by * in **(Be)**, arrows in **(Bf)** indicate strongly PAS stained bodies.

Total RNA was isolated from stems of 4 individual 3 years old *A. tequilana* plants dissected as described in Figure 3A or whole rhizome tissue (Figure 1Ab), using the TRIzol reagent (Invitrogen) and the PureLink RNA mini Kit (Invitrogen) according to the manufacturers protocol. cDNA templates for qRT-PCR were synthesized by incubating 1 μg of total RNA with a mix of specific reverse primers and the revertaid reverse transcriptase (Thermo Scientific) according to the manufacturer’s instructions. Each cDNA was prepared in duplicate. The genes selected for the analysis were the fructosyltransferase isoforms *Atq1-SST2, Atq6G-FFT1*, *Atq6G-FFT2* and the fructan exo-hydrolase isoform *AtqFEH4* using primers described in Avila de Dios et al., 2015. qRT-PCR was carried out by using Kapa Sybr Fast qPCR master mix (2X) Universal reagent (Sigma Aldrich) in a StepOne Plus thermocycler (Applied Biosystems, Foster City, CA, United States) under the following thermal conditions: 20 s at 95°C followed by a total of 40 cycles of 3 s at 95°C and 30 s at 60°C, followed by the Melt curve stage in order to determine specificity of RT-PCR amplicons with continual fluorescence acquisition during a 65–95°C thermal period. Relative transcript abundance was normalized and calculated by using Ubiquitin11 (*AtqUBI11*). Data shown describes mean values of relative quantification obtained from two independent amplification reactions and the error bars indicate the standard deviation of the mean. The analysis was performed by using the software StepOne v2.3 (Applied Biosystems) and the relative quantification was determined by using the equation according to the ΔΔCT mathematical method. Amplification efficiency (90–110%) for each primer set was determined by amplification of targets of a cDNA dilution series (1:5).

## Results

### Non-structural Carbohydrate Distribution in the Agave Stem and Rhizome

**FIGURE 2:**
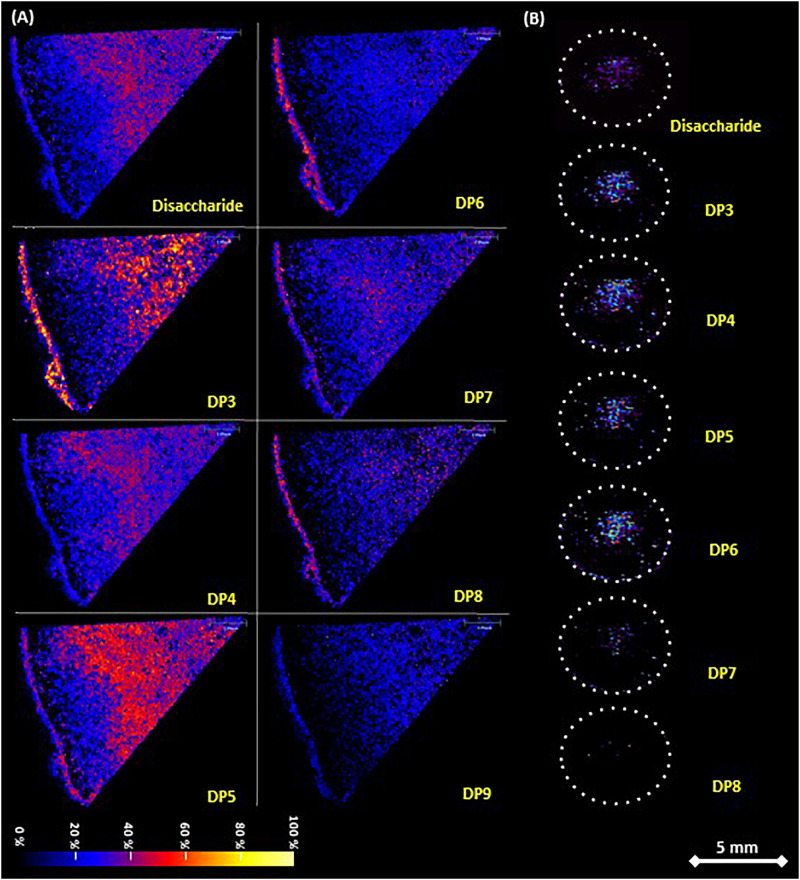
Fructan distribution in *A. tequilana* stem and rhizome tissue determined by MALDI-MSI on tissue printed nylon membrane. **(A)** Stem sections as shown in Figure 1Aa, sample sprayed matrix and analyzed in SYNAPT G2 spectrometer. Detection of disaccharide and fructooligosaccharides from DP3 to DP9 is indicated. **(B)** Rhizome sections as shown in Figure 1Ab, sublimated matrix and run in AB equipment. Dotted circles indicate the periphery of the sections. Detection of disaccharide and fructooligosaccharides from DP3 to DP8 is indicated. Both adducts [M+Na]^+^ and [M+K]^+^ were merged for each disaccharide to fructan DP9 while abundance is shown as a change in color intensity. Scale bar is 5 mm.

Staining of transverse and longitudinal sections of *A. tequilana* stem and rhizome tissue (Figure 1Aa) using PAS (a general method for detection of carbohydrates) or Lugol reagent (starch specific), as shown in Figure 1Ba–f confirmed that, starch synthetizing/accumulating cells are restricted to a layer that encompasses the stem at the primary growth meristem region, just below the leaves as shown by the thin black line indicating lugol staining around the periphery (Figure 1Bb). These data suggest that PAS staining in the central stem region is due to the accumulation of fructans or sucrose rather than starch. Higher magnification shows strong PAS staining in vascular tissue, in the apoplast, at the periphery of vacuoles and at specific points within the vacuole (Figure 1Bc,d). This indicates more specific localization of fructans at the subcellular level. PAS staining of emerging roots and rhizomes (Figure 1Ab and Supplementary Figures 1G,H) showed an intense coloration indicating the probable accumulation of fructans in this rapidly growing tissue and revealed strong staining in the central core of the rhizome which is comprised of a large number of vascular bundles as shown in Figure 1Be. A closer examination of tissue between the vascular bundles (Figure 2Bf) also shows strong PAS staining concentrated in yet unidentified bodies close to the cell membrane or within the apoplast. This is the first time an association between high levels of carbohydrates and the rhizome core harboring vascular tissue has been reported in *A. tequilana* and supports the hypothesis that carbohydrates (fructans) are mobilized from the mother plant through the rhizome to provide nutrients for the emerging and rapidly growing offsets (asexual reproduction form) which arise from rhizomes. However, PAS staining is limited to the detection of carbohydrates in specific tissues but provides no information on the levels or types of carbohydrate molecules (such as sucrose or fructans) present.

**FIGURE 3:**
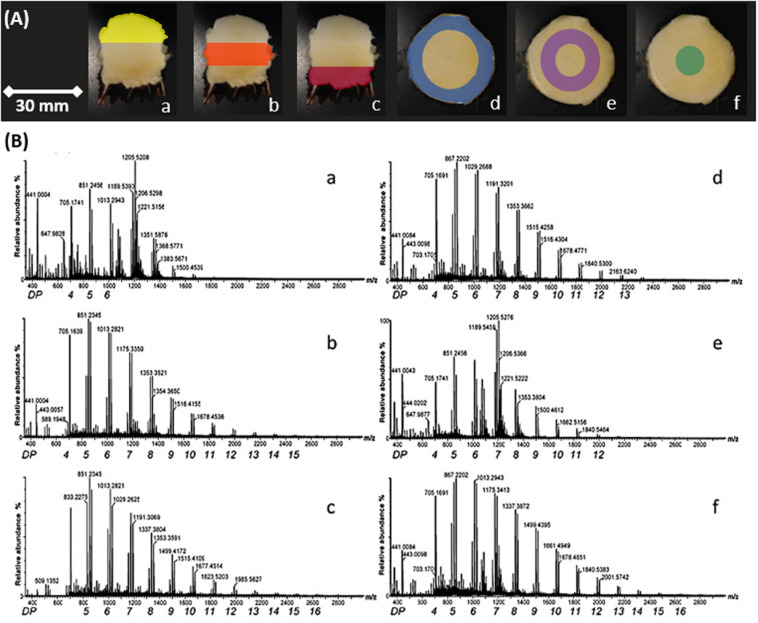
MS spectra of fructans from *A. tequilana* stem sections. **(A)** Examples of transverse and longitudinal sections of *A. tequilana* stem tissue. Colors indicate different dissections of the stem from which fructans were extracted. **(B)** Positive ionization mass spectra of fructans of spotted extracts from *A. tequilana* stem in relation to the dissected areas shown in **(A)**. Similar spectra were generated for a total of four individual plants, the image shown is representative of the data obtained.

In order to address this, the distribution of fructan polymers in *A. tequilana* stem and rhizome tissue, was analyzed by MALDI-IMS-MSI to obtain a label-free detection of the relative abundance and distribution of fructans *in situ*. To achieve this a novel tissue-printing method was developed as described in methods (Supplementary Figure 1). The amount of carbohydrates retained *in situ* on the nylon membrane was adequate to carry out mass spectrometry imaging analysis and the tissue printed membranes could therefore be used to determine the relative abundance and distribution of fructan polymers over the stem tissue section (Figure 2A). Tissue printed images of transverse and longitudinally dissected stem tissue developed using the MSI technique, confirmed the localization of fructans determined by PAS in the center of the stem tissue surrounded by a peripheral “fructan-free” zone (a dark ring around the periphery of the stem) (Figure 2), corresponding to the layer of starch (Figure 1Bb). Images obtained by MSI tissue printing of rhizome tissue (Figure 2B) are also consistent with the PAS staining of rhizomes (Figure 1Bf) where fructans are localized in the highly vascularized central core of the rhizome consistent with mobilization of fructans to developing offsets.

### Detailed Fructan Profiling and Location

Inspection of the MALDI images in relation to specific DP of the fructan molecules showed a gradient of complexity from the periphery of the stem toward the center (Figure 2A) and from the shoot apical meristem (SAM) toward the root (Supplementary Figure 1H), although a mixture of fructans of varying DPs is observed toward the center of the stem, the total amount of fructan does not significantly differ (Supplementary Figure 3A). In the rhizome core fructans of DP 3-6 were most abundant, consistent with the mobilization hypothesis where (smaller) lower DP fructans would be most effectively mobilized within the vascular system.

In order to confirm the fructan content and DP levels across the stem and rhizome tissue, samples were obtained from transversely and longitudinally dissected stem tissue and transversely dissected rhizome tissue from 3 years old *A. tequilana* plants as indicated in Figures 1A, 3A, and Supplementary Figure 1. DP levels across the stem and rhizome extracts were also analyzed by MS and the data are consistent with the MSI analysis. Figure 3Ba–f, shows positive ionization mass spectra of oligo-fructan extracts spotted onto a MALDI plate from tissues described in Figure 3A. Oligo-fructans of DP 4–7 were the most abundant in all samples and in stem tissue immediately below the SAM, (Figure 3Aa yellow) where only oligo fructans (with DP ≤ 7) were observed. The central and lower stem longitudinal sections showed similar profiles with DP up to 17 (Figure 3Ab,c orange and red). In rhizome tissue, a disaccharide was the most abundant form (Supplementary Figure 2) and the presence of fructans of DP3-8 was confirmed. Enzymatic quantification of fructans and thin layer chromatography (TLC) profiles, of stem and rhizome tissue (Supplementary Figure 3B) showed that overall fructan content in rhizome tissue is lower than in stem tissue also consistent with the putative functions of these organs where stem tissue is the main long term storage organ for carbohydrates whereas the rhizome functions as the conduit between the storage organ and newly developing plantlets (Figure 1Ab).

The stem cross sections also show fructans of up to DP15 in both the most peripheral (Figure 3Ad blue) and the most internal stem sections (Figure 3Af green), whereas the intermediate section (Figure 3Ae purple) shows abundance of DP7 fructans, and up to DP12. The innermost section (Figure 3Bf) also shows a higher relative abundance of DP 8–17 in comparison to sections d and e. These data indicate that although a general gradient of increasing DP can be observed from the periphery to the core and from the SAM to the root, the intermediate regions (Figure 3Bb,e) are more heterogeneous.

### Ion Mobility Spectrometry-Mass Spectrometry (IMS-MS) Enables Fructan Isoform Detection

**FIGURE 4:**
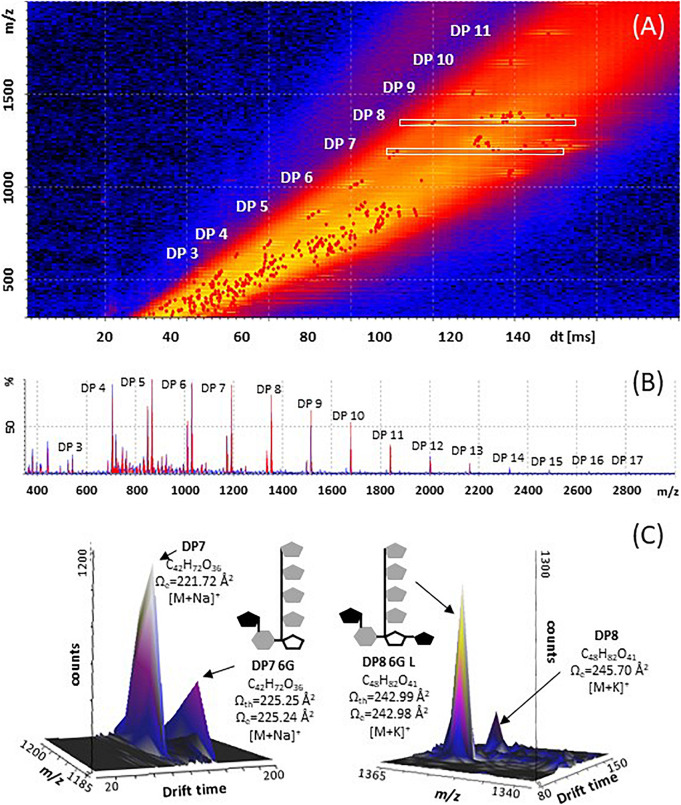
Structural diversity of fructans in *A. tequilana* stem (3 years old plants), using Mass Spectrometry Imaging and Ion Mobility Mass Spectrometry. **(A)** Mobilogram plotted in DriftScope 2.8 (Waters Corporation, Milford, MA, United States), red dots are ions with a specific x: drift time [ms], and y: m/z ratio **(B)** MALDI mass spectrum, in red fructan ions of plot A with their relative abundances. **(C)** 3D Plot mobilogram of observed isoforms and predicted isoform structures (x: drift time [ms], y: signal intensity [counts], z: *m/z* ratio, a zoom into DP7 [M+Na]^+^ and DP8 [M+K]^+^, where experimental CCS values (Ω_*e*_) of fructans, m/z and dt data were obtained with a SYNAPT G1 using poly-(D/L)-alanine as calibrant, and N_2_ as drift gas. Cartoon structures are drawn based in theoretical CCS values (Ω_*th*_) calculated for energy minimized isoforms.

To determine in more detail the nature of the fructan molecules in different sections of *A. tequilana* stem tissue, ion mobility spectrometry-mass spectrometry (IMS-MS) was carried out. The technique can serve a two-fold purpose: add a separation dimension that is partly orthogonal to mass spectrometry and determine molecular structure (Ridenour et al., 2010). In this study, IMS-MS was used for fructan isoform discrimination and in combination with mass spectrometry imaging a relationship between location and structural conformations of the molecules could be determined (Figures 4A–C). Calculation of experimental collisional cross sections (CCS) for fructans was also carried out. The CCS values were derived using Poly-(D/L)-alanine for calibration of mobility since although they are from a distinct molecular class in comparison to fructans, they cover the same mass-to-charge ratio (*m/z*). Obtained experimental CCS calibration data in comparison with previously reported CCS values for poly-(D/L)-alanine oligomers showed an adjustment well below 3.0% as relative standard deviation (RSD).

The CCS values presented here are the first approximation for fructans from agave stem analyzed on both tissue printed samples and aqueous extracts, however, Poly-(D/L)-alanine oligomers have linear structures, whereas agavins are complex branched molecules, and so far, none of the available standards adequately fit their structural characteristics. Here, a calibration using poly-(D/L)-alanine was carried out that allowed CCS values with an RSD < 3.0%, to be obtained, when comparing with CCS values from databases (Supplementary Table 1). For agave fructans formation of both sodium and potassium adducts was observed (Table 1). As an example, Figure 4C, shows isoforms of DP7 and DP8 with a theoretical structure predicted by molecular modeling. Two isoforms were detected in agave stem tissue for fructans of DP 4, 5, 6, 7, 8, 10, and 11. Interestingly experimental CCS values for all stem fructan isoforms derived with Poly-(D/L)-alanine calibration displayed smaller CCS values compared to the same DP inulin isoforms (Table 1), suggesting that fructans from agave stems could potentially be more compact structures than linear inulin isoforms.

**TABLE 1:** Experimental Collision Cross Section (CCS) values for agave fructans of duplicate samples derived from ion mobility data obtained with a SYNAPT G1 mass spectrometer previously calibrated with poly-(D/L)-alanine using Nitrogen as drift gas.

Fructan DP	*m/z*	CCS values (Å^2^)				
		Inulin†	Agave stem isoforms			
			Average^*T*^	StdDev	Average^*T*^	StdDev
DP3 [M+Na]^+^	527.1786	132.43	135.67	0.13	n.d.	n.d.
DP3 [M+K]^+^	543.1529	149.91	136.43	0.17	n.d.	n.d.
DP4 [M+Na]^+^	689.2372	164.25	163.22	0.39	n.d.	n.d.
DP4 [M+K]^+^	705.2118	164.98	162.81	0.42	184.98	0.49
DP5 [M+Na]^+^	851.2964	188.35	182.97	0.56	n.d.	n.d.
DP5 [M+K]^+^	867.2710	190.32	186.36	0.86	188.87	0.29
DP6 [M+Na]^+^	1013.3557	207.56	202.67	0.14	205.48	0.32
DP6 [M+K]^+^	1029.3308	210.17	n.d.	n.d.	n.d.	n.d.
DP7 [M+Na]^+^	1175.4151	230.57	221.72	0.65	225.24	0.94
DP7 [M+K]^+^	1191.3899	226.69	224.47	0.74	228.03	0.97
DP8 [M+Na]^+^	1337.4740	248.65	240.63	0.77	243.52	0.35
DP8 [M+K]^+^	1353.4489	248.07	242.98	0.53	245.70	0.44
DP9 [M+Na]^+^	1499.5372	271.98	262.15	0.21	n.d.	n.d.
DP9 [M+K]^+^	1515.5084	271.58	262.65	0.82	n.d.	n.d.
DP10 [M+Na]^+^	1661.5910	294.05	282.56	0.46	293.76	0.38
DP10 [M+K]^+^	1677.5656	294.73	282.39	0.67	289.73	0.78
DP11 [M+Na]^+^	1823.6491	316.99	301.82	0.28	n.d.	n.d.
DP11 [M+K]^+^	1839.6233	311.66	301.11	0.90	311.53	0.50
DP12 [M+Na]^+^	1986.7109	332.11	n.d.	n.d.	n.d.	n.d.
DP12 [M+K]^+^	2001.7110	n.d.	n.d.	n.d.	n.d	n.d.

*^*dagger*^Analytical standard solution spotted on the MALDI plate. ^*T*^Average of duplicates. n. d., no detected.*

**FIGURE 5:**
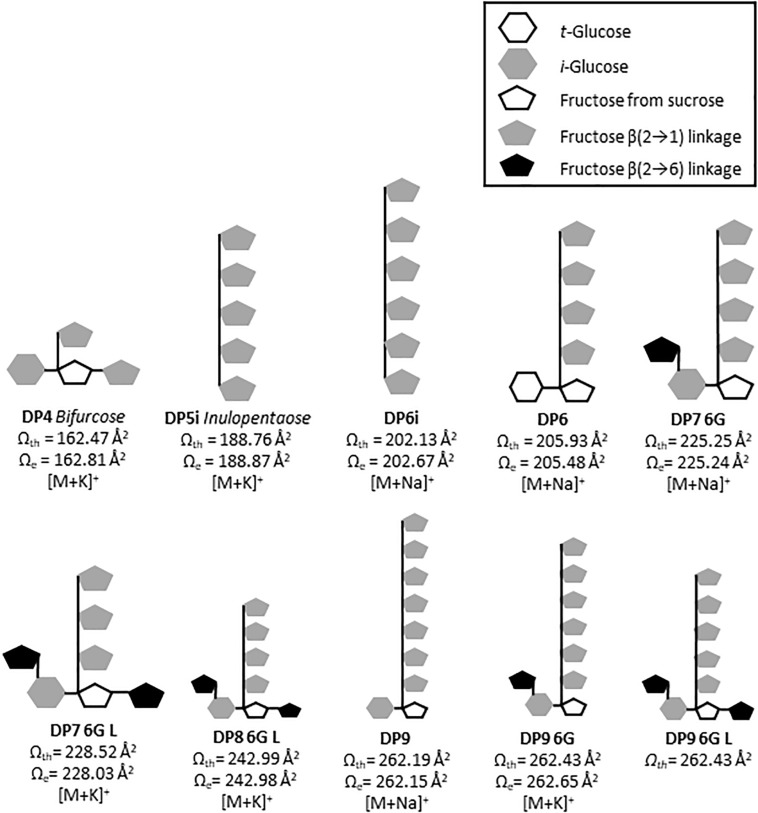
Putative predicted structures of fructans identified in *A. tequilana* stem. Cartoon structures are drawn based in theoretical CCS values (Ω_*th*_) calculated for energy minimized isoforms. Where G refers to glucose, i inulin and L levan type fructan.

In order to identify the most probable structures for the detected isoforms, theoretical CCS values were calculated considering all 3-dimensional structures (Supplementary Figures 4A–D) and all possible conformations as described in section “Materials and Methods.” Table 2 shows experimental and calculated CCS values for all possible isoforms from DP4 to DP11. Data with asterisk indicate candidate putative structures for experimentally determined CCS values and as can be observed, whereas only one structure is consistent with data for molecules of DP 4, 5, 6, and 7, two or three possible structures are consistent with data obtained for fructans of DP 8 and 9, respectively. From each derived CCS value, a structural conformation for the fructan molecules was predicted by molecular modeling as shown in Figure 5. To validate our predictions we plotted the experimental CCS values (CCS_*e*_) vs. theoretical CCS values (CCS_*th*_) for inulin fructans from DP3 to DP11 and found a linear correlation between CCS_*e*_ and CCS_*th*_ with an *R*^2^ value of 0.992 and 0.993 when comparing [M+Na]^+^ and [M+K]^+^ adducts, respectively, with CCS_*th*_ for inulins of same DPs (Supplementary Figure 6). A comparison between CCS_*e*_ and CCS_*th*_ showed on average a deviation below 3.5% between both values.

**TABLE 2:** Experimental and theoretical calculated CCS values for agave stem fructans.

DP	Adduct	Experimental		Calculated CCS values Ω_*th*_ (Å)^2^										
		CCS (Å)^2^												
**4**	[M+Na]^+^	163.22	–	DP4	DP4i	DP4-6G	DP4-6GG	DP4-L	DP4L2					
	[M+K]^+^	162.81*	184.98	160.74	160.83	157.6	156.91	162.47*	155.32					
**5**	[M+Na]^+^	182.97	–	DP5	DP5i	DP5-6G	DP5-6GG	DP5-6G-L	DP5-6G-L2					
	[M+K]^+^	186.36	188.87*	178.07	188.76*	172.93	175.68	183.21	178.6					
**6**	[M+Na]^+^	202.67*	205.48**	DP6	DP6i	DP6-6G	DP6-6GG	DP6-6G-L	DP6-6G-L2					
	[M+K]^+^	–	–	205.93**	202.13*	194.48	189.42	193.31	199.08					
**7**	[M+Na]^+^	221.72	225.24*	DP7	DP7i	DP7-6G	DP7-6GG	DP7-6G-L	DP7-6G-L2	DP7-6GG-L	DP7-6GG-L2			
	[M+K]^+^	224.47	228.03**	215.54	232.2	225.25*	205.24	228.52**	227.12	207.03	213.75			
**8**	[M+Na]^+^	240.63	243.52	DP8	DP8i	DP8-6G	DP8-6GG	DP8-6G-L	DP8-6G-L2	DP8-6GG-L	DP8-6GG-L2			
	[M+K]^+^	242.98*	245.70	238.16	242.19	231.03	238.64	242.99*	238.39	225.45	237.91			
**9**	[M+Na]^+^	262.15*	–	DP9	DP9i	DP9-6G	DP9-6GG	DP9-6G-L	DP9-6G-L2	DP9-6GG-L	DP9-6GG-L2			
	[M+K]^+^	262.65**	–	262.19*	257.76	262.43**	251.14	262.43**	257.8	251.11	253.33			
**10**	[M+Na]^+^	282.56	293.76	DP10	DP10i	DP10-6G	DP10-6GG	DP10-6G-L	DP10-6G-L2	DP10-6GG-L	DP10-6GG-L2	DP10-6G-L2-L2	DP10-6GG-L2-L2	
	[M+K]^+^	282.39	289.73	271.83	278.04	265.85	277.58	285.09	286.62	272.18	263.33	264.95	264.95	
**11**	[M+Na]^+^	301.82	–	DP11	DP11i	DP11-6G	DP11-6GG	DP11-6G-L	DP11-6G-L2	DP11-6GG-L	DP11-6GG-L2	DP11-6G-L2-L2	DP11-6GG-L2-L2	DP11-L2-L2
	[M+K]^+^	301.11	311.53	291.58	298.44	278.9	281.87	294.42	282.99	289.67	283.49	283.18	296.46	298.72

DP4i: “i” means Inulo-tetraose, or glucose-free oligosaccharide, so that PD5i means inulopentaose or 5 fructose residues β(2→1)-linked. See Supplementary Figure 4A–D for more structural information. Data values with * and ** means calculated CCS_*th*_ that are similar to CCS_*e*_, so that potential structure are predicted from calculation of CCS_*th*_.

**FIGURE 6:**
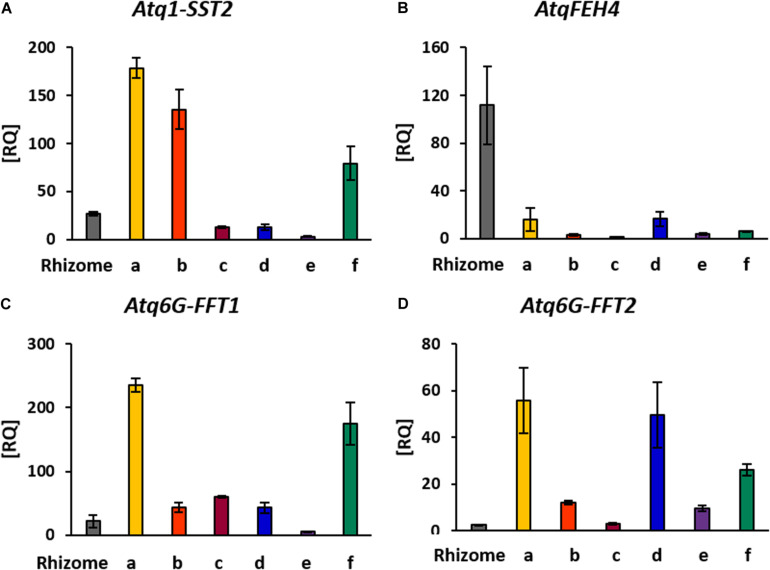
RT-PCR Expression analysis of genes involved in fructan metabolism. **(A)** *Atq1-SST2*: sucrose-sucrose 1 fructosyl transferase isoform 2, **(B)** *AtqFEH4*: fructosyl exo-hydrolases isoform 4, **(C,D)** *Atq6G-FFT1/2*: fructan-fructan 6G fructosyltransferases isoforms 1 and 2. Rhizome tissue is indicated and all other tissues correspond to stem dissections indicated in Figure 3A.

Synthesis of agavins and fructans of different complexity requires the coordination of enzyme activities specific for the synthesis or degradation of the different glycosidic linkages involved and of the regulation of the encoding genes. To determine whether the expression patterns of genes encoding enzymes involved in fructan metabolism were consistent with the localization of fructans of different complexities in stem and rhizome tissue, qRT-PCR analysis (Figure 6) was carried out for genes encoding fructan synthesizing enzymes: *Atq1-SST2*, *Atq6G-FFT* (1 and 2) and a fructan degrading enzyme, *AtqFEH4*. *Atq1-SST2* was expressed in all tissue sections although most strongly in the uppermost [shoot apical meristem (SAM)] region (Figure 3Aa). Both *Atq6G-FFT1* and 2 were also most strongly expressed in tissue close to the SAM (Figure 3Aa) whereas *Atq6G-FFT1* was strongly expressed in the center of the stem (section f), in contrast *Atq6G-FFT2* was strongly expressed in peripheral stem tissue (section d) and *AtqFEH4* was only expressed at low levels in peripheral stem tissue (section d) but highly expressed in rhizome samples.

## Discussion

In many plant species, fructans accumulate in specialized storage organs such as tubers, corms or bulbs (Hendry, 1993). However, when the supply of substrates exceeds the demand of sucrose, fructan accumulation can also occur in leaves, stems, roots, and other non-reserve organs (Stumpf and Conn, 1980; Peukert et al., 2014; Cimini et al., 2015). In *Agave tequilana*, fructans are found in almost all plant organs including leaves, rhizomes and floral tissue, although the main storage organ is the stem where complex, branched agavin molecules have been identified (Mellado-Mojica and López, 2012). Evidence for the roles of fructans in signaling, stress tolerance and osmoregulation are increasing, and they are thought to be key metabolites for the adaptation of *Agave* species to hot arid environments. In order to understand the many putative functional roles of fructans in agaves, it is necessary to determine the localization and abundance of different types of fructan both within specific tissues and at the subcellular level. PAS staining showed the presence of fructans in the vacuole as previously described (Mellado-Mojica et al., 2016) and the higher magnification images presented here suggest that they are not distributed evenly but accumulate around the periphery and in concentrated points within this organelle. PAS staining also showed the presence of significant levels of carbohydrates in the apoplast of stem tissue and agave apoplast fluid has been shown previously to contain fructans (Wang and Nobel, 1998). This observation is consistent with proposed roles for fructans in stress responses and signaling. It is often assumed that nutrients are transported from agave mother plants to developing offsets through the rhizomes, in this case PAS staining and MSI of significant levels of carbohydrates associated with vascular tissue in the rhizome core support this hypothesis but does not reveal the nature of the carbohydrate molecules.

Previous methods used to determine the presence of fructans are destructive involving extraction of fructans from sampled tissue and making the spatial distribution of different fructan polymers difficult to discern. The relatively novel methodology of MALDI-MSI has previously been applied to determine fructan presence in barley grains by analyzing tissue sections (Peukert et al., 2014). Recently, (Witzel and Matros, 2020) also applied MALDI-MSI analysis of tissue sections to determine fructan distribution in *Asparagus officinalis* L., a close relative of *Agave* species. Preparation of tissue sections, however, can be time consuming and good quality sections of some tissues such as fibrous agave stems are often difficult to acquire and can only be carried out over relatively small areas, besides soluble metabolites, as fructans, can be lost during sample processing. To facilitate the MALDI-MSI analysis for agave stem and rhizome tissue, we developed a tissue printing method using nylon membrane, a durable material with higher binding capacity than nitrocellulose and polyvinylidene difluoride (PVDF). The tissue printed samples have several advantages since they can be easily produced, stored and transported between laboratories. The method could easily be adapted to cover larger areas and distinct tissues of agaves, or other plant species, kinds of metabolites and imprinting surfaces (Bjarnholt et al., 2014; Dong et al., 2016).

The results obtained by MALDI-MSI of tissue printed *A. tequilana* stems show a gradient of increasing fructan DP from the periphery of the stem toward the core which confirm the exclusion of fructans from the starch layer just below the leaves at the primary growth meristem (PGM) (Zavala-García et al., 2018). The PGM is the region of expansion of the stem and from where leaf primordial initiates and grows. Starch storage in this region is probably transient in order to supply carbohydrates for these growing tissues and excess carbohydrate (sucrose) can be converted to fructan for long-term storage in the stem. The presence of low DP fructans at the stem periphery is consistent with this hypothesis and as the stem tissue grows and ages toward the core, more complex agavins are synthesized. Interestingly, for the first time a strong accumulation of fructans was also observed in the primordia of developing roots or rhizomes and specifically in the central vascular tissue of the rhizome. Witzel and Matros, 2020, report an increasing fructan DP and complexity toward the root tissues surrounding but not within the vascular system in asparagus. This contrasts with the distribution observed for agave rhizome tissue where only the central vascular tissue shows an accumulation of fructans. The comparison between the *Asparagus* and *Agave* genera (both members of the Asparagaceae family) shows contrasting patterns of fructan accumulation in relation to tissue type and potential function. Whereas in *A. tequilana*, fructans provide a long-term carbohydrate reservoir in stem tissue, the same function may be accomplished by fructan storage in roots of *A. officinalis*. High levels of low DP fructans within the vascular region of rhizome tissue in *A. tequilana* are consistent with roles in water retention/osmoregulation and carbohydrate mobilization for rapid growth and production of offsets rather than long term carbohydrate storage, as in the agave stem.

Previous characterization of *A. tequilana* stem fructan structures revealed a complex mixture consisting of a combination of graminans and neo-fructan structures called agavins (Lopez et al., 2003), or as inulin and neo-fructan chains (Mancilla-Margalli and López, 2006; Arrizon et al., 2010; Praznik et al., 2013), which also contain internal and external glucose units (Mancilla-Margalli and López, 2006). Fructan DP variations in *A. tequilana* have also been identified in many studies, which can range from 3 to 30 with both β(2→1) and β(2→6) glycosidic linkages and it has been reported that the degree of polymerization can reach 70, depending on the age and the developmental stage of plants analyzed (Arrizon et al., 2010; Praznik et al., 2013). This structural diversity has been studied by several techniques including nuclear magnetic resonance (NMR), high performance liquid chromatography (HPLC), gas chromatography mass spectrometry (GC-MS), among others (Mancilla-Margalli and López, 2006; Arrizon et al., 2010; Montañez-Soto et al., 2010; Praznik et al., 2013; Acosta-Domínguez et al., 2018).

The MALDI-IMS-MSI approach is a general strategy for determination of relative abundance, location and structure of label-free metabolites (Hove et al., 2010; Kaspar et al., 2011; Bjarnholt et al., 2014; Heyman and Dubery, 2016), neither extraction nor sample processing are need, where analysis occurs simultaneously within the same sample and in this study, provided insights into size, shape, diversity and distribution of fructans in the central stem of *Agave tequilana*. Using a simple sample preparation method (Goodwin, 2012; Dong et al., 2016), MS analysis was suitable for fructan analysis where matrix peaks (less than ∼350 *m/z*) did not overlap with the molecules of interest.

For the first time ion mobility data were obtained for agave stem fructans, allowing the measurement of the rotationally averaged collision cross-section (CCS) for fructans of DP 3–12 with detection of both sodium [M+Na]^+^ and potassium [M+K]^+^ adducts, providing specific information according to the collisional cross section of a molecule in the gas phase (Paglia et al., 2014). CCS values were derived in order to detect structural differences between molecules of the same *m/z* ratio that suggest different 3D conformations. The structures predicted by molecular modeling confirm that in agave stems a range of DP isoforms occur including DP4 as bifurcose, DP5i and DP6i as inulopentaose and inulohexaose, respectively, and DP6 with an external glucose as previously reported (Mellado-Mojica and López, 2012). More complex structures have also been identified from our analysis such as two isomers of DP7-6G or neoheptaose, one isoform of DP8 and three possible isoforms of DP9. A detailed representation of the DP7-6G structure is shown in Supplementary Figure 5 as an example. Previous studies have reported isoforms in *A. tequilana* stems, up to DP5 (Mellado-Mojica and López, 2012) and DP 6 (Praznik et al., 2013). Due to technical limitations and lack of standards, non-structural assignation has been done for molecules of DP > 6. Therefore, IMS-MS and computational modeling for fructan (up to DP 11), are proposed here as a promising approximation which will complement chemical analysis.

Matrix-assisted laser desorption/ionization mass spectrometry analysis of agave fructans in positive mode detected mainly sodium and potassium adducts. The highest DP observed in agave fructan extracts was DP18 (Figure 3B), all fructan DP detected were validated by tandem mass spectrometry (MS/MS, Supplementary Figure 7), whereas MSI revealed the presence of oligomers up to DP17 (Figure 4B), with up to two isoforms per DP in stems of 3 years old plants. Additionally, separation of isomeric ions by IMS-MS, also enabled the identification of fructans up to DP17, with two isoforms detected for some DPs (Table 1). Future studies should incorporate MALDI-IMS-MSI in negative ionization mode in order to compare experimental CCS values for deprotonated ions, as recent evidence suggested that sodium adducts for oligosaccharides could potentially provoke glycan species to adopt a more compact structure with the cation fully solvated by carbohydrate OH groups. Considerable improvements for ion mobility separation of deprotonated species could therefore be achieved (Struwe et al., 2016). More work also needs to be done in terms of computational modeling to calculate theoretical CCSs values. Our results show a basic modest structural modeling using lower energy conformers to predict theoretical CCS values, by the projection approximation method (the most basic procedure). Further calculations should incorporate more accurate geometry optimization methods, such as density functional theory as well as use of more accurate methods to estimate theoretical CCS values, such as for example, the trajectory method (one of the most accurate methods).

In contrast to the electrospray ionization method, singly charged ions were detected in fructans using MALDI imaging on a nylon tissue printed sample. Several studies carried out using LC-MS in positive ionization mode have reported multiply charged ions in fructans from ryegrass of up to DP100, where 1 to 5 charged ions were observed (Harrison et al., 2011). Malto-oligosaccharides up to DP35 have also been detected as triply charged ions (Rashid et al., 2014), and more recently pectic oligosaccharides have been separated using ion mobility mass spectrometry and up to DP8 have been measured with quadruply charged ions detected (Leijdekkers et al., 2015). Lopez et al., 2003 have also reported fructans from DP3 to DP29 in an 8 years old agave plant, and from DP 3 to DP30 have been identified in a 4 years old plant using MALDI-ToF-MS in positive ionization mode (Arrizon et al., 2010).

The identification of complex agavin structures, implies a coordinated and strict regulation of the enzymes involved in fructan metabolism. Although four distinct enzyme activities are necessary for the synthesis of complex agavins, only two different enzyme types have been reported to date based on transcriptome data (Avila de Dios et al., 2015). Expression of genes encoding enzymes involved in fructan metabolism is modified in presence of elicitors (Suárez-González et al., 2016); and it is thought that enzymes involved in fructan synthesis may carry out multiple activities as has been shown for onion bulbs (Ritsema et al., 2003). A first step to determine the regulatory mechanisms underlying fructan metabolism is to establish whether there is a correlation between the expression patterns of the genes encoding enzymes involved in fructan metabolism and fructan localization and molecular structure. Strong expression of *Atq1-SST2* in peripheral stem tissue where sucrose transported from the leaves will be converted to fructan is consistent with its role in synthesis of the most basic fructan trisaccharide, 1-kestotriose from which more complex molecules are derived. Expression of *Atq6G-FFT* (1 and 2) in peripheral and internal tissues is also consistent with their roles in the synthesis of more complex fructan molecules and the possibility that both *Atq6G-FFT* might have multiple activities. Interestingly the *AtqFEH4* gene is most strongly expressed in rhizome tissue and to a lesser extent in stem tissue. This result is consistent with a high fructan turnover in rhizome tissue leading to the mobilization of low DP fructans or disaccharide products of fructan hydrolysis in order to support the growth and development of new offsets.

## Data Availability Statement

The original contributions presented in the study are included in the article/Supplementary Material, further inquiries can be directed to the corresponding author.

## Author Contributions

JS and JO-O conceived the study. JO-O and JS coordinated the investigation. AP-L performed the MS analysis. MC supported the MS analysis with SYNAPT G2 and AB instruments. JO-O supported the MS analysis with SYNAPT G1. AP-L performed fructan quantifications, histology analysis, and sample preparations. AG-V carried out expression analysis. JO-O carried out CCS computational calculations. All authors contributed to the final version and read the final manuscript.

## Conflict of Interest

The authors declare that the research was conducted in the absence of any commercial or financial relationships that could be construed as a potential conflict of interest.

## Supplementary Material

Supplementary Figure 1 Outline of tissue printing technique. **(A)** *Agave tequilana* plant showing the crown region (leaf/stem to root transition). Dotted red line indicates the longitudinal axis, dotted blue line indicates the transversal axis. **(B)** *A. tequilana* plant as in **(A)** with leaves and most roots removed and dissected longitudinally. **(C)** Longitudinal stem section, **(D)** Transverse stem section. **(E)** Representation of tissue printing process. **(F)** Tissue printed transverse section mounted on MALDI Imaging plate. **(G)** PAS staining of a tissue printed longitudinal section. **(H)** MALDI-ToF-MSI of a tissue printed longitudinal section obtained, using a sprayer for matrix application and a QTOF SYNAPT G1 spectrometer with a spatial resolution of 100 μm per pixel.

Supplementary Figure 2 MALDI positive ionization mass spectrum of fructans of spotted extracts from *A. tequilana* rhizome as shown in Figure 1Ab.

Supplementary Figure 3 Total fructan content of *A. tequilana* stem and rhizome sections. **(A)** Percentage of fructan mg/g in total carbohydrate extracts of fresh tissue. Rhizome tissue is indicated and all other tissues correspond to stem dissections indicated in Figure 3A. **(B)** Thin layer chromatography of fructan extracts. Std, standards; G, glucose; F, fructose; S, sucrose; K, kestotriose; N, nystose; and FN, fructofuranosyl nystose. Bar errors correspond to standard deviation.

Supplementary Figure 4 **(A)** Possible DP6 fructan isoforms. **(B)** Possible DP7 fructan isoforms. **(C)** Possible DP8 fructan isoforms. **(D)** Possible DP10 and 11 fructan isoforms.

Supplementary Figure 5 Representations of DP7-6G structure.

Supplementary Figure 6 Inulin structures and linear regression of CCS_*e*_ and CCS_*th*_.

Supplementary Figure 7 MSMS fragmentation data and MSMS spectra of fructans DP3 to DP18.

Supplementary Table 1 Collision cross-sections (Å^2^) for singly charged protonated oligomers of poly-(D/L)-alanine measured in nitrogen as drift gas.
