# Supplementary figures and images for: Localization and Composition of Fructans in Stem and Rhizome of Agave tequilana Weber var. azul
Source: Front Plant Sci. 2021 Jan 20;11:608850. doi: 10.3389/fpls.2020.608850 (PMC7855178; doi:10.3389/fpls.2020.608850)

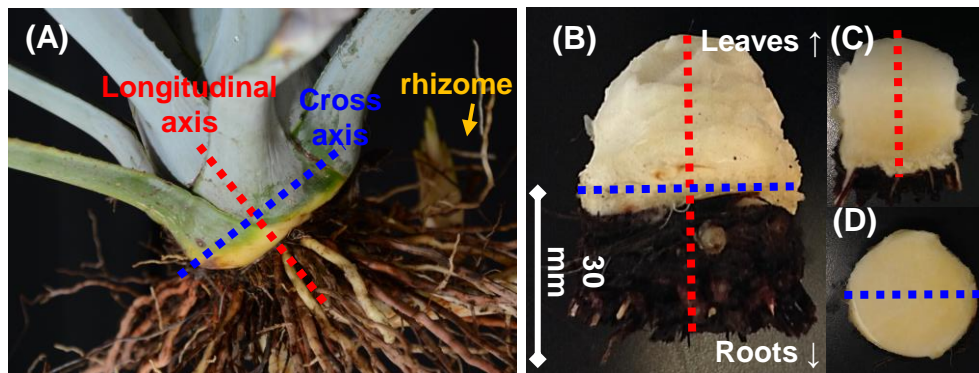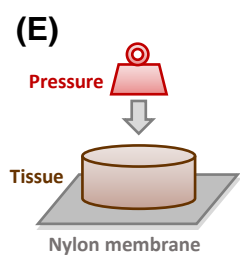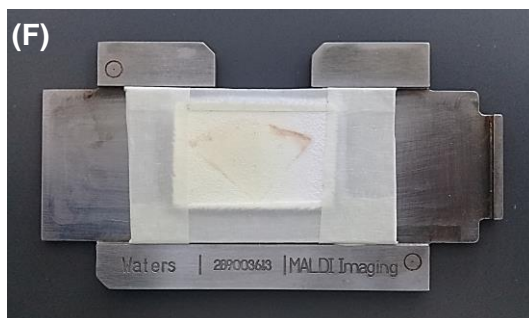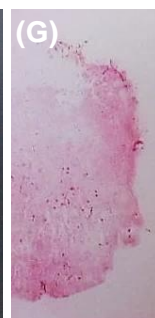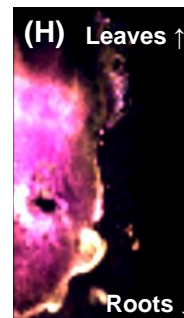

Supplement: Supplementary Figure 1 — Outline of tissue printing technique. (A) Agave tequilana plant showing the crown region (leaf/stem to root transition). Dotted red line indicates the longitudinal axis, dotted blue line indicates the transversal axis. (B) A. tequilana plant as in (A) with leaves and most roots removed and dissected longitudinally. (C) Longitudinal stem section, (D) Transverse stem section. (E) Representation of tissue printing process. (F) Tissue printed transverse section mounted on MALDI Imaging plate. (G) PAS staining of a tissue printed longitudinal section. (H) MALDI-ToF-MSI of a tissue printed longitudinal section obtained, using a sprayer for matrix application and a QTOF SYNAPT G1 spectrometer with a spatial resolution of 100 μm per pixel. [file Image_1.pdf]

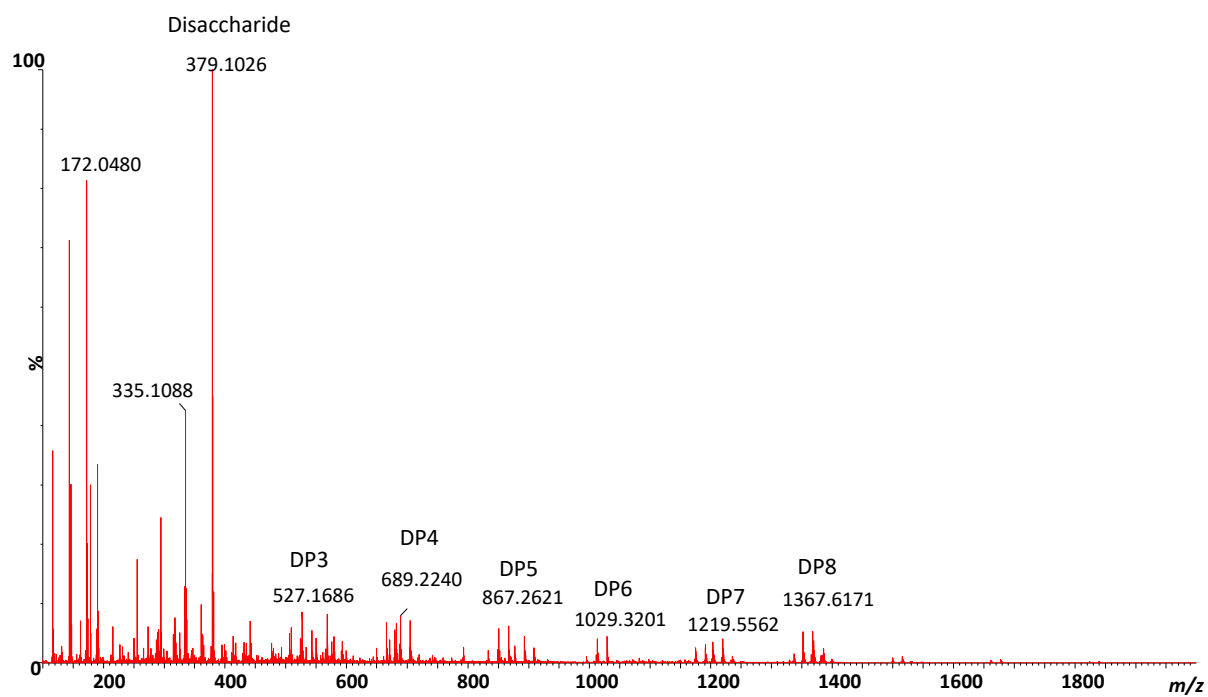

Supplement: Supplementary Figure 2 — MALDI positive ionization mass spectrum of fructans of spotted extracts from A. tequilana rhizome as shown in Figure 1Ab. [file Image_2.pdf]

(A)

Fructan % [w/w]

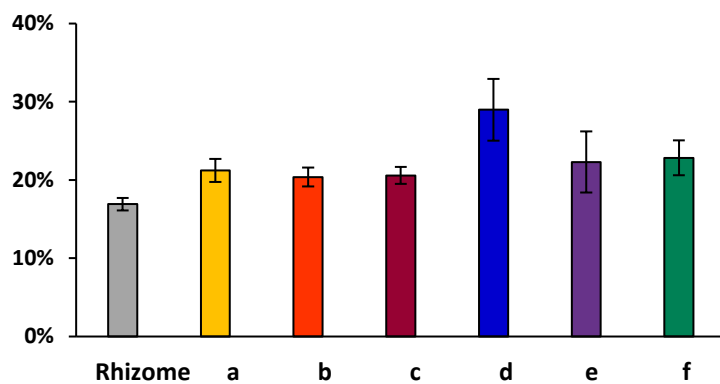

(B)

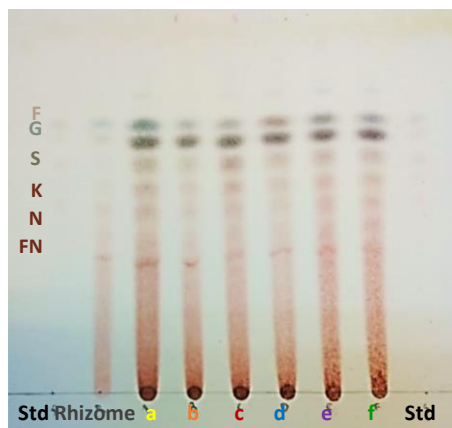

Supplement: Supplementary Figure 3 — Total fructan content of A. tequilana stem and rhizome sections. (A) Percentage of fructan mg/g in total carbohydrate extracts of fresh tissue. Rhizome tissue is indicated and all other tissues correspond to stem dissections indicated in Figure 3A. (B) Thin layer chromatography of fructan extracts. Std, standards; G, glucose; F, fructose; S, sucrose; K, kestotriose; N, nystose; and FN, fructofuranosyl nystose. Bar errors correspond to standard deviation. [file Image_3.pdf]

Figure S4A

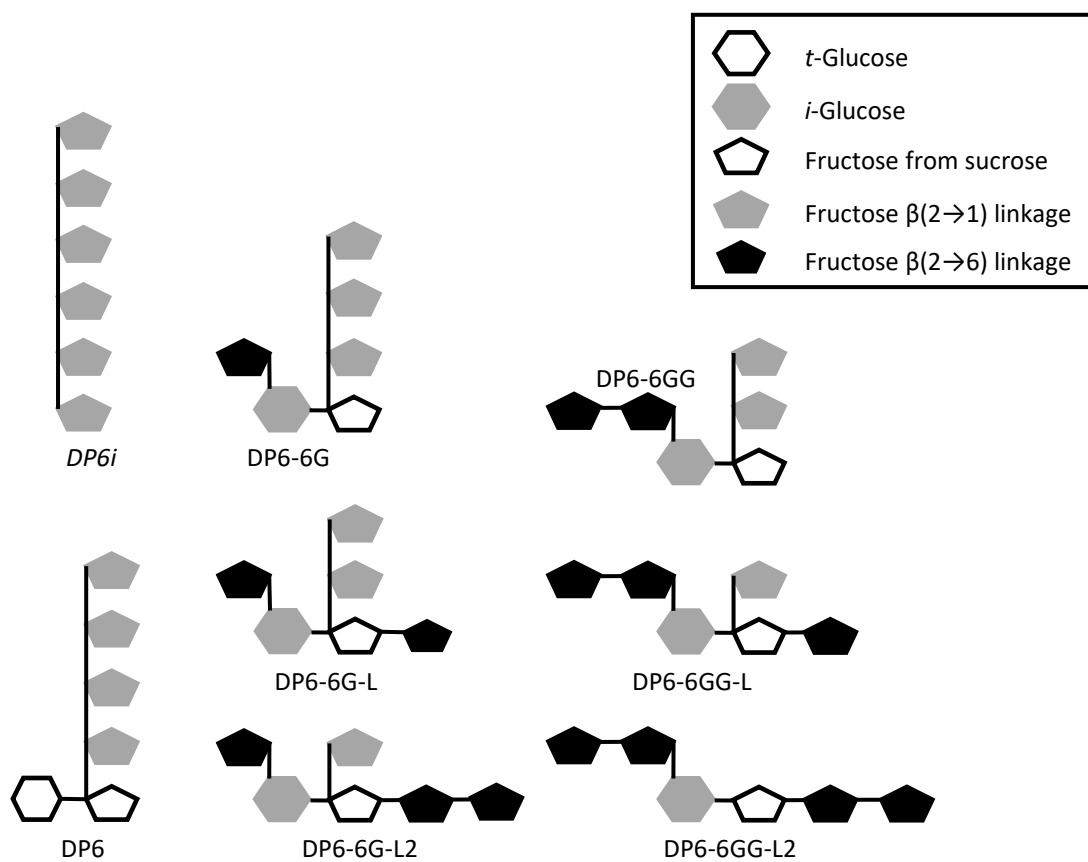

Figure S4B

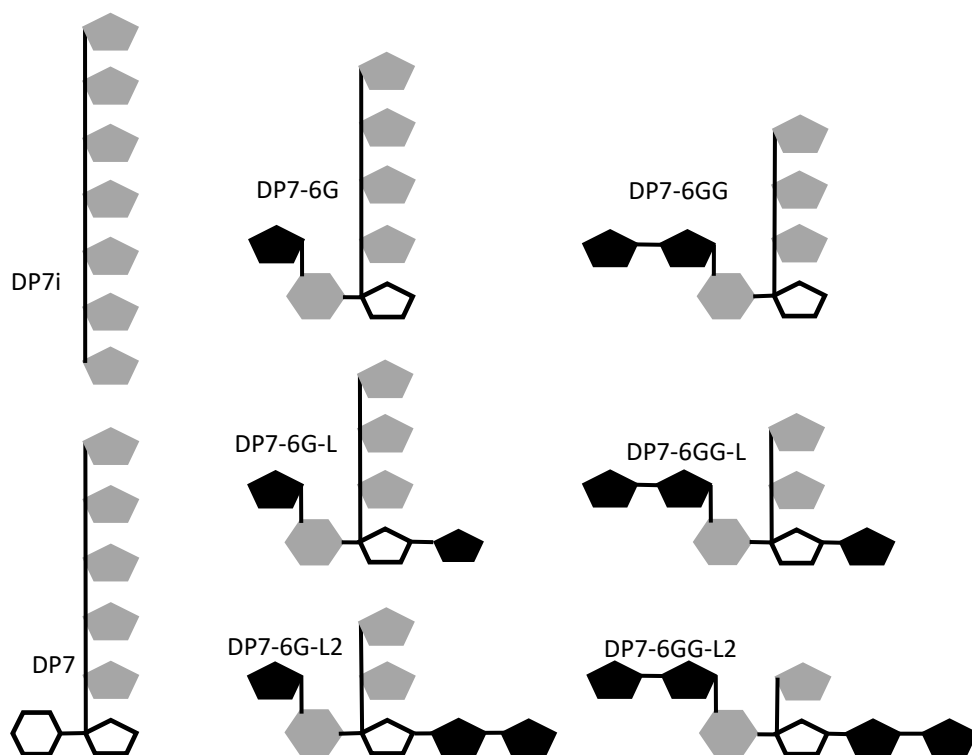

Figure S4C

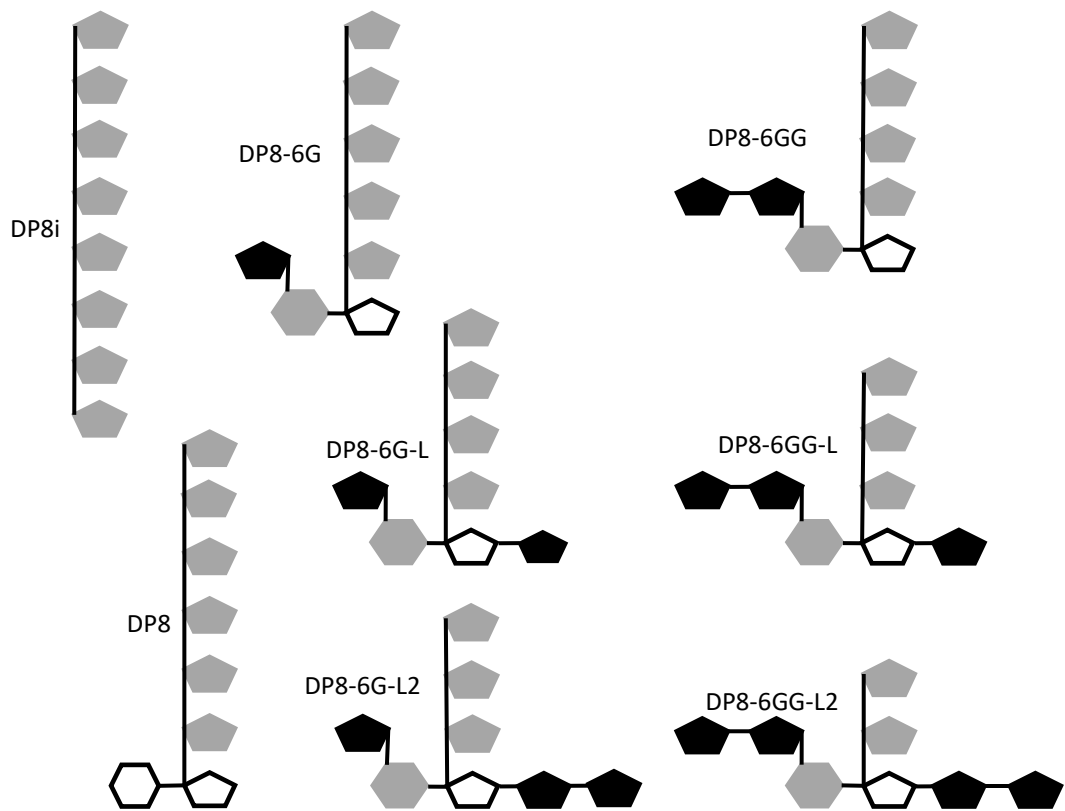

Figure S4D

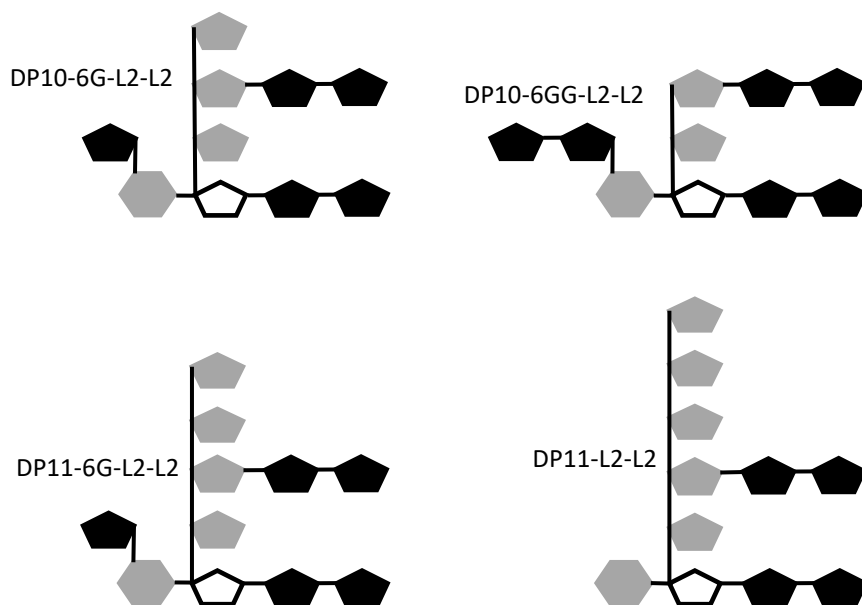

Supplement: Supplementary Figure 4 — (A) Possible DP6 fructan isoforms. (B) Possible DP7 fructan isoforms. (C) Possible DP8 fructan isoforms. (D) Possible DP10 and 11 fructan isoforms. [file Image_4.pdf]

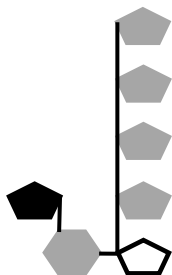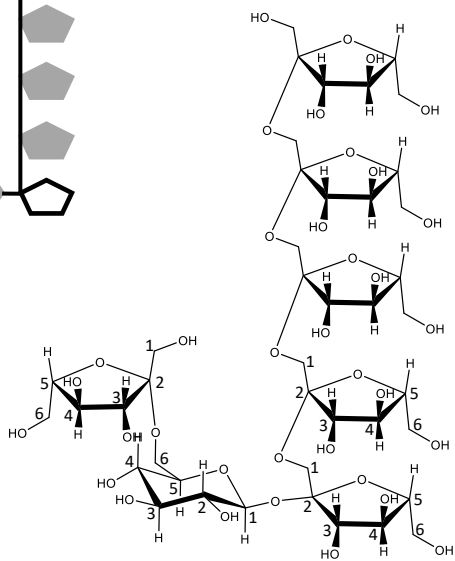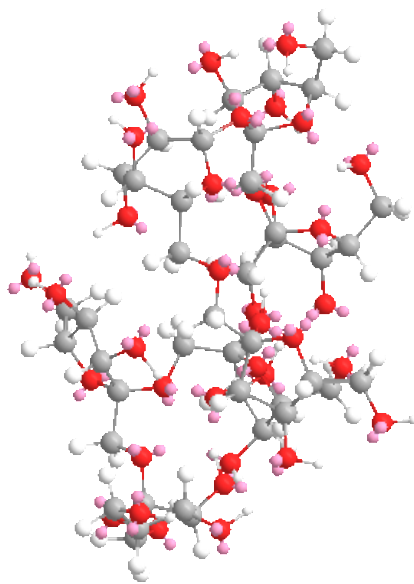

$$\Omega_{th} = 225.25 \text{ \AA}^2$$

Supplement: Supplementary Figure 5 — Representations of DP7-6G structure. [file Image_5.pdf]

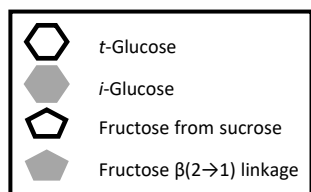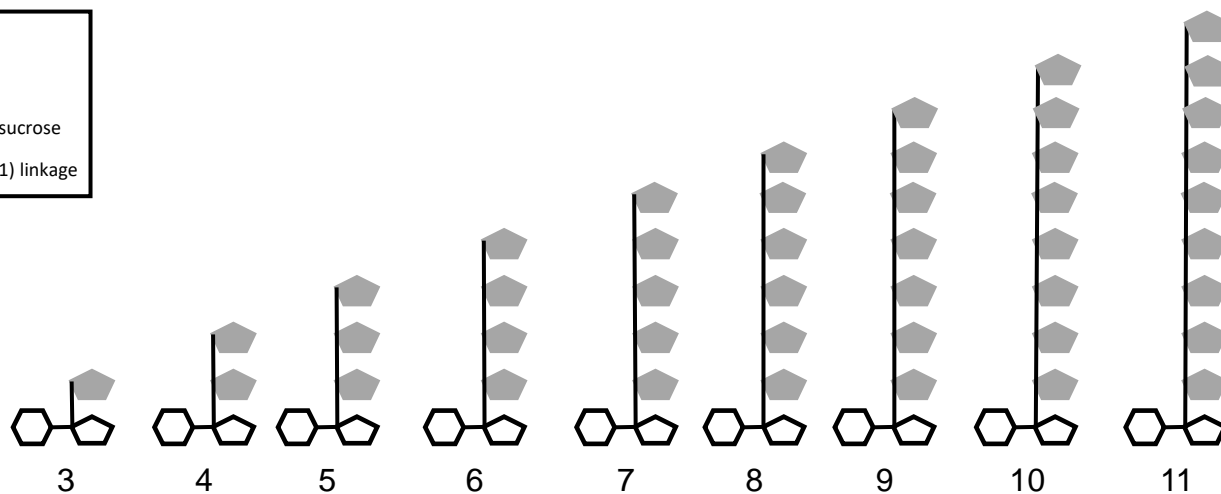

| DP                             | 3      | 4      | 5      | 6      | 7      | 8      | 9      | 10     | 11     |
|--------------------------------|--------|--------|--------|--------|--------|--------|--------|--------|--------|
| $CCS_e [M+Na]^+ \text{ \AA}^2$ | 132.43 | 164.25 | 188.35 | 207.56 | 230.57 | 248.65 | 271.98 | 294.05 | 316.99 |
| $CCS_e [M+K]^+ \text{ \AA}^2$  | 149.91 | 164.98 | 190.32 | 210.17 | 226.69 | 248.07 | 271.58 | 294.73 | 311.66 |
| $CCS_{th} \text{ \AA}^2$       | 140.51 | 160.74 | 178.07 | 205.93 | 215.54 | 238.16 | 262.19 | 271.83 | 291.58 |

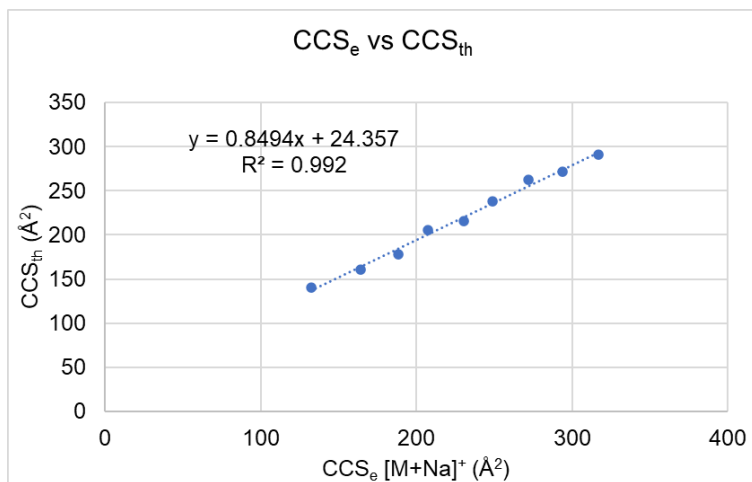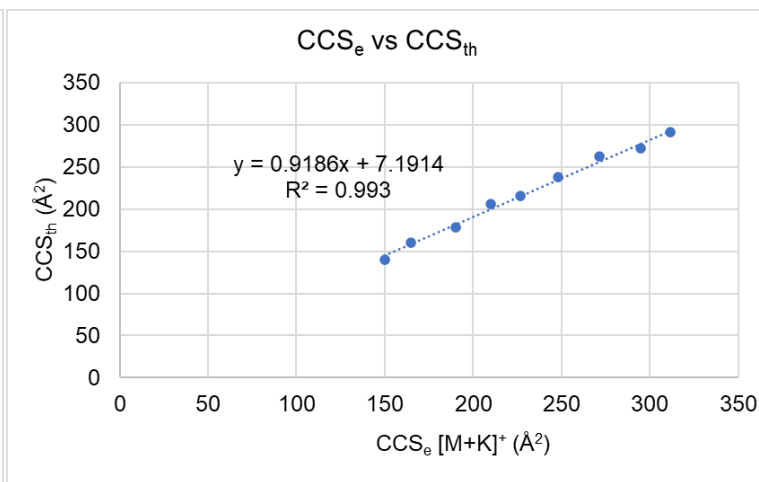

Supplement: Supplementary Figure 6 — Inulin structures and linear regression of CCSe and CCSth. [file Image_6.pdf]
