# Supplementary material for: Localization and Composition of Fructans in Stem and Rhizome of Agave tequilana Weber var. azul
Source: Front Plant Sci. 2021 Jan 20;11:608850. doi: 10.3389/fpls.2020.608850 (PMC7855178; doi:10.3389/fpls.2020.608850)

Detected fragment ions in MSMS fragmentation of fructans from DP3 to DP18

Fructan DP	Formula	Monoistopic Mass M	*m/z*			
			M [+Na]	M [+K]	M-H2O [+Na]	M-H2O [+K]
**n.d.**	$C_7H_{12}O_7$	208,0583	231,0481	247,022	213,0375	229,0114
**n.d.**	$C_8H_{13}O_7$	221,0661	244,0559	260,0298	226,0453	242,0192
**n.d.**	$C_{10}H_{11}O_6$	227,0556	250,0454	266,0193	232,0348	248,0087
**n.d.**	$C_8H_{13}O_8$	237,0610	260,0508	276,0247	242,0402	258,0141
**n.d.**	$C_9H_{14}O_9$	266,0638	303,0692	319,0431	285,0586	301,0325
**n.d.**	$C_{10}H_{16}O_9$	280,0794	289,0536	305,0275	271,043	287,0169
**n.d.**	$C_{10}H_{16}O_5$	296,0743	319,0641	335,038	301,0535	317,0274
**n.d.**	$C_{12}H_{22}O_{11}$	342,1162	365,1060	381,0799	347,0954	363,0693
**n.d.**	$C_{14}H_{21}O_{12}$	381,1033	404,0931	420,067	386,0825	402,0564
**DP3**	$C_{18}H_{32}O_{16}$	504,1690	527,1588	543,1327	509,1482	525,1221
**DP4**	$C_{24}H_{42}O_{21}$	666,2219	689,2117	705,1856	671,2011	687,175
**DP5**	$C_{30}H_{52}O_{26}$	828,2747	851,2645	867,2384	833,2539	849,2278
**DP6**	$C_{36}H_{62}O_{31}$	990,3275	1013,3173	1029,2912	995,3067	1011,2806
**DP7**	$C_{42}H_{72}O_{36}$	1152,3800	1175,3698	1191,3437	1157,3592	1173,3331
**DP8**	$C_{48}H_{82}O_{41}$	1314,4330	1337,4228	1353,3967	1319,4122	1335,3861
**DP9**	$C_{54}H_{92}O_{46}$	1476,4860	1499,4758	1515,4497	1481,4652	1497,4391
**DP10**	$C_{60}H_{102}O_{51}$	1638,54	1661,5298	1677,5037	1643,5192	1659,4931
**DP11**	$C_{66}H_{112}O_{56}$	1800,5916	1823,5814	1839,5553	1805,5708	1821,5447
**DP12**	$C_{72}H_{122}O_{61}$	1962,6444	1985,6342	2001,6081	1967,6236	1983,5975
**DP13**	$C_{78}H_{132}O_{66}$	2124,6973	2147,6871	2163,661	2129,6765	2145,6504
**DP14**	$C_{84}H_{142}O_{71}$	2286,7501	2309,7399	2325,7138	2291,7293	2307,7032
**DP15**	$C_{90}H_{152}O_{76}$	2448,8029	2471,7927	2487,7666	2453,7821	2469,756
**DP16**	$C_{96}H_{162}O_{81}$	2610,8557	2633,8455	2649,8194	2615,8349	2631,8088
**DP17**	$C_{102}H_{172}O_{86}$	2772,9086	2795,8984	2811,8723	2777,8878	2793,8617
**DP18**	$C_{108}H_{182}O_{91}$	2934,9614	2957,9512	2973,9251	2939,9406	2955,9145

Tandem mass spectrum MS^2 of DP3 ion over a spotted extract of agave stem.

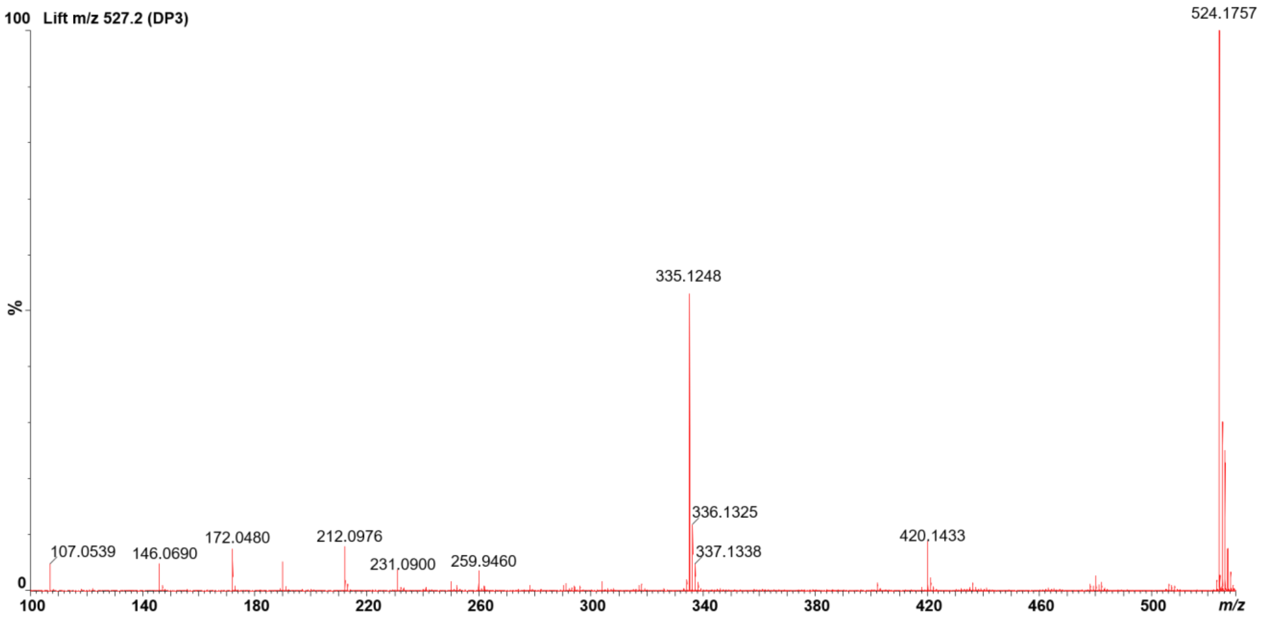

Tandem mass spectrum MS^2 of DP4 ion over a spotted extract of agave stem.

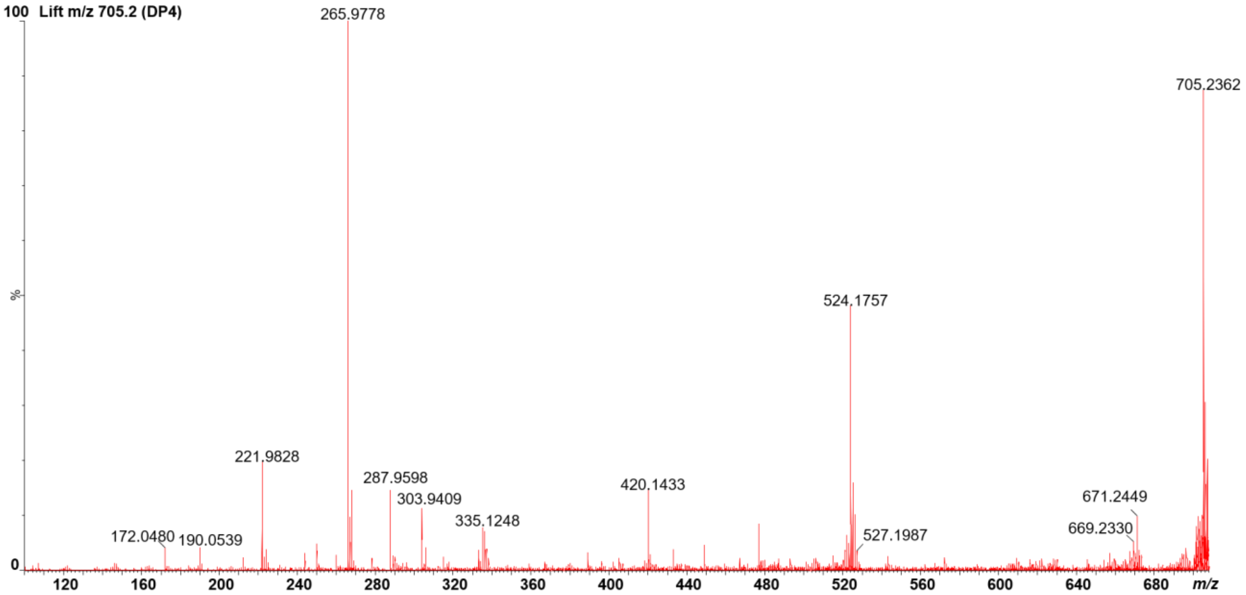

Tandem mass spectrum MS^2 of DP5 ion over a spotted extract of agave stem.

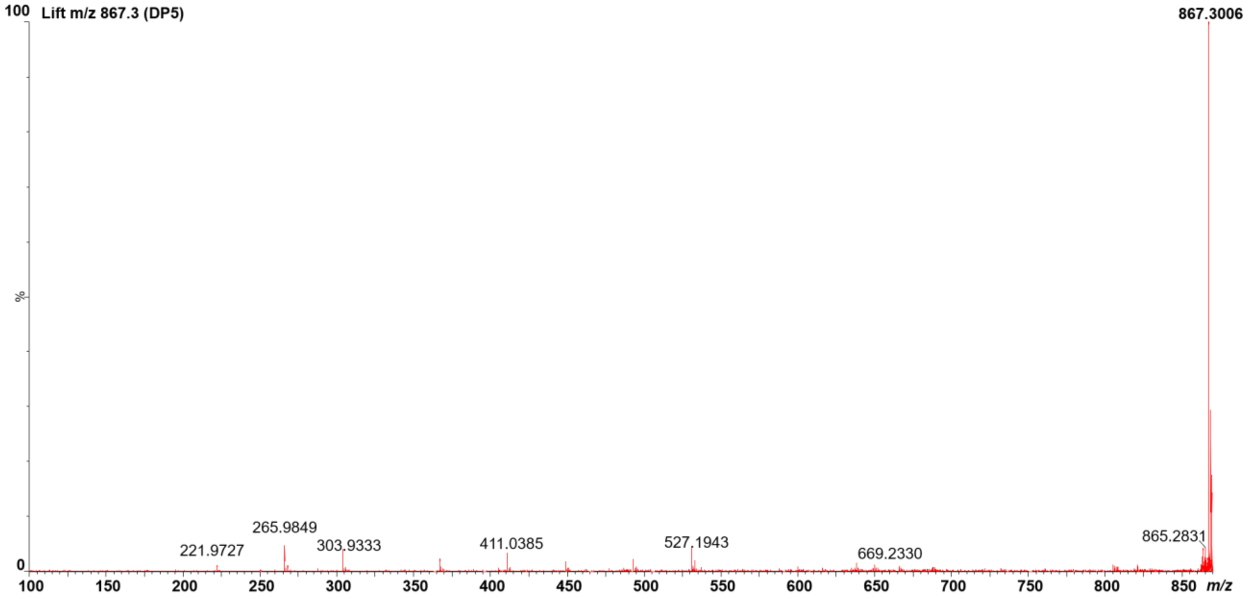

Tandem mass spectrum MS^2 of DP6 ion over a spotted extract of agave stem.

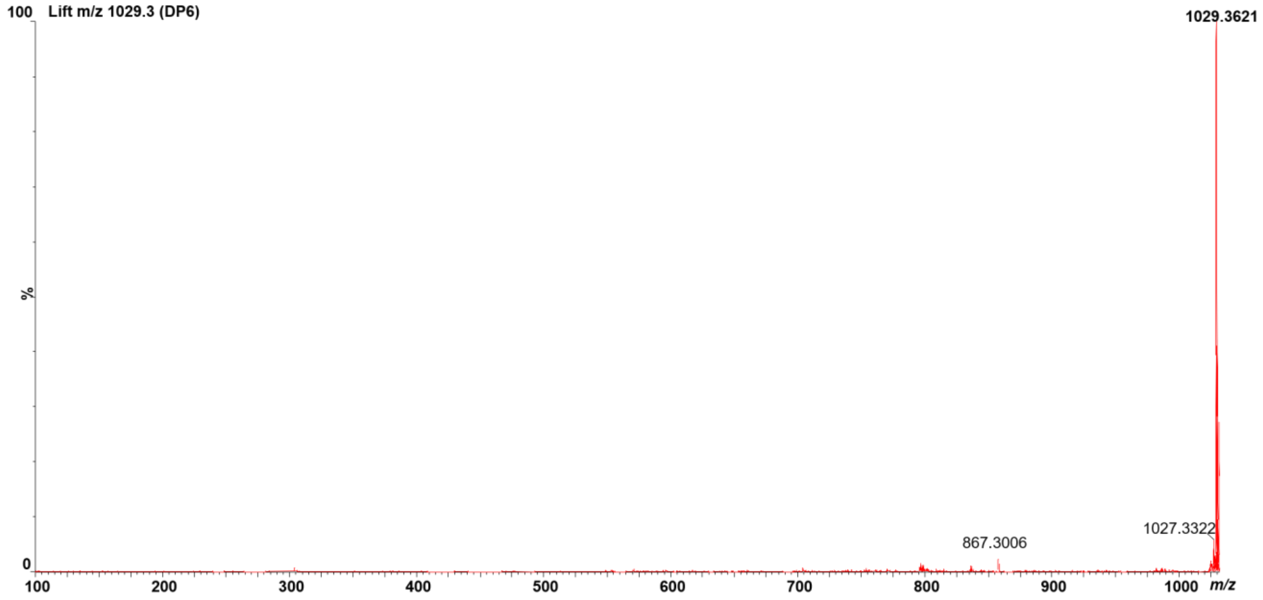

Tandem mass spectrum MS^2 of DP7 ion over a spotted extract of agave stem.

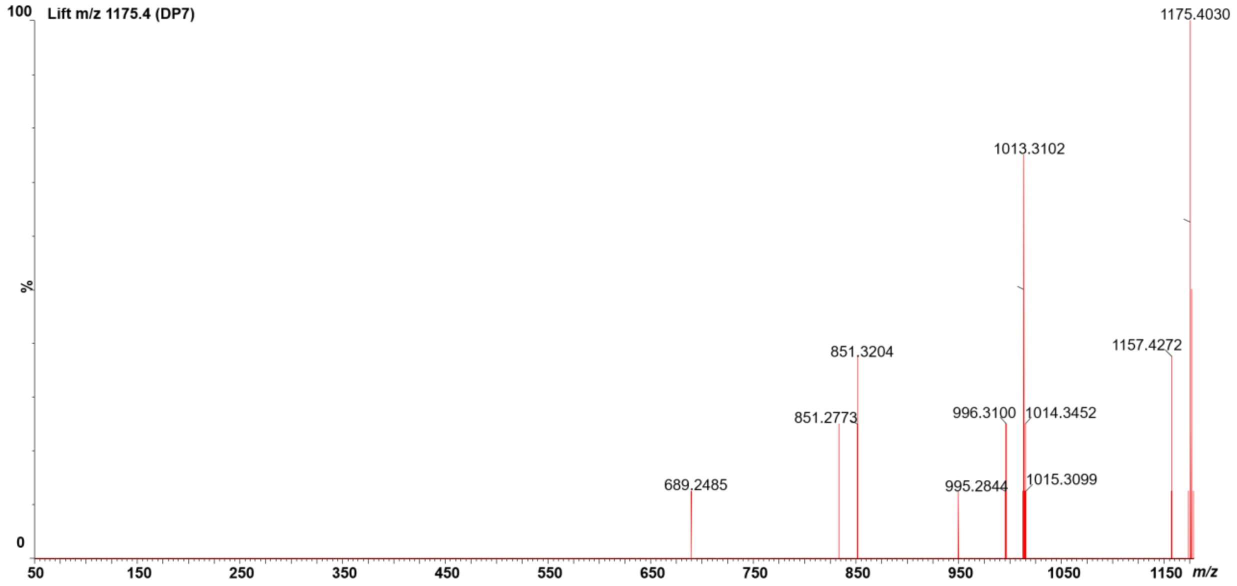

Tandem mass spectrum MS^2 of DP8 ion over a spotted extract of agave stem.

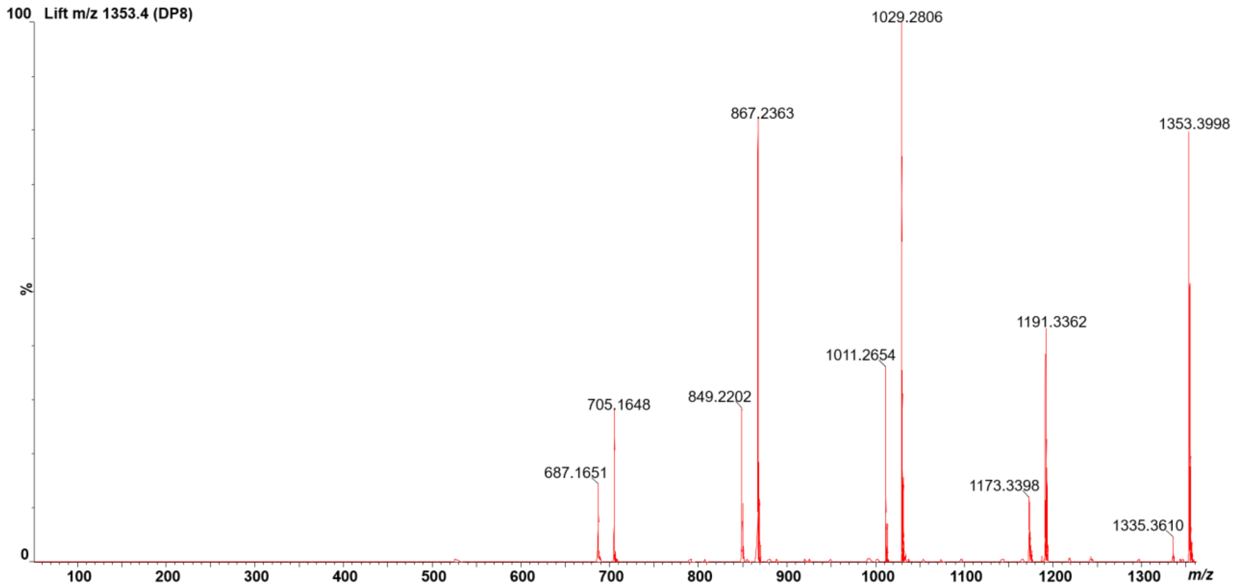

Tandem mass spectrum MS^2 of DP9 ion over a spotted extract of agave stem.

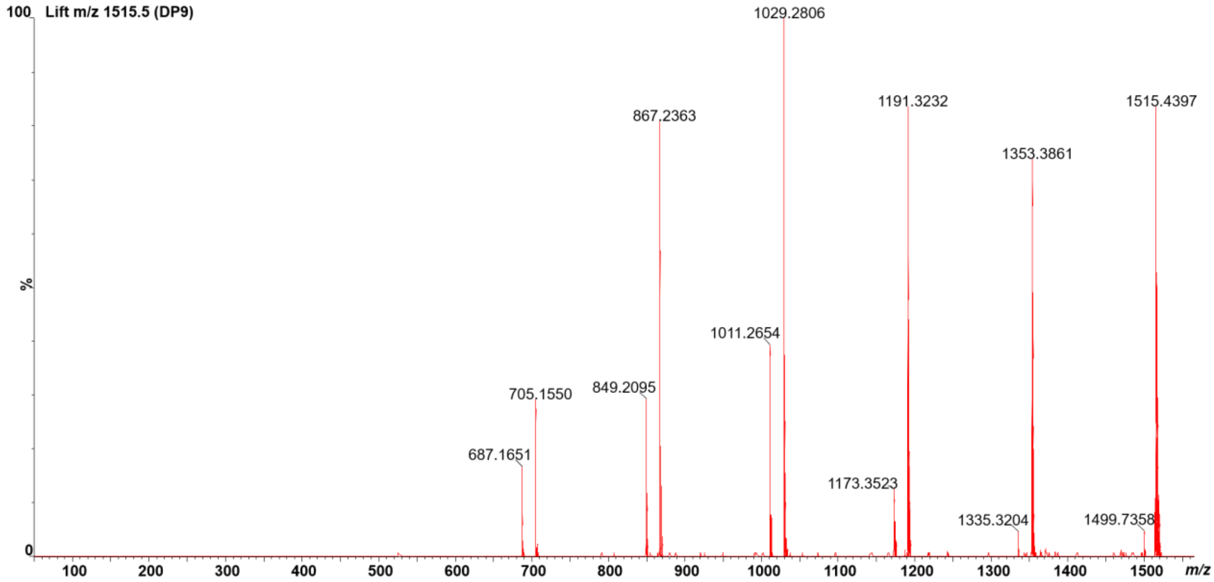

Tandem mass spectrum MS^2 of DP10 ion over a spotted extract of agave stem.

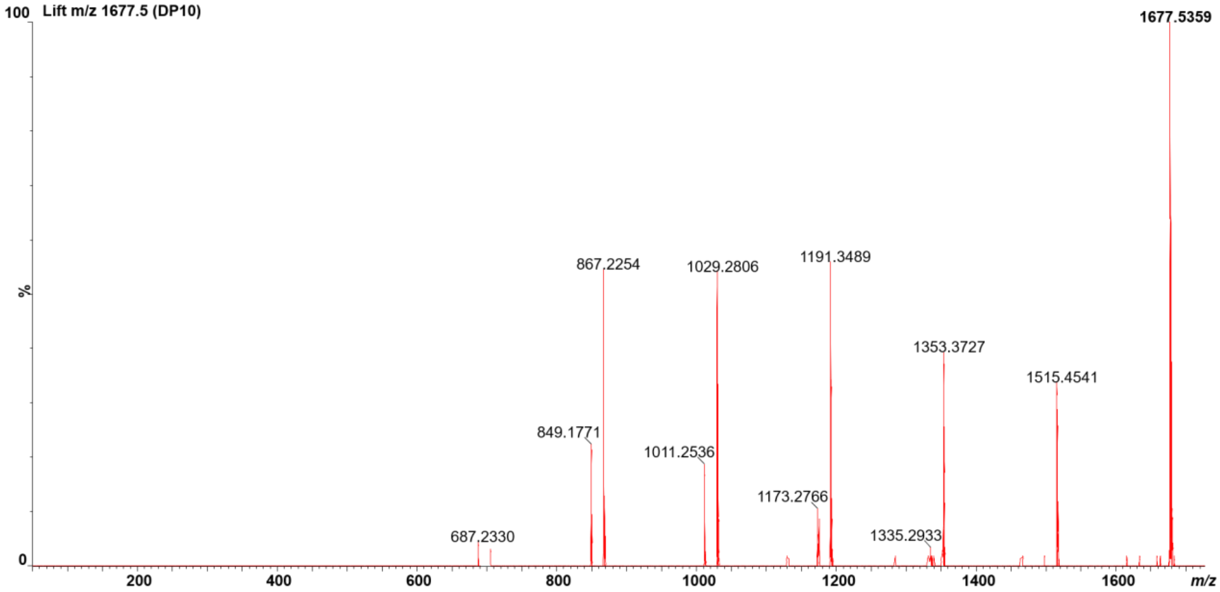

Tandem mass spectrum MS^2 of DP11 ion over a spotted extract of agave stem.

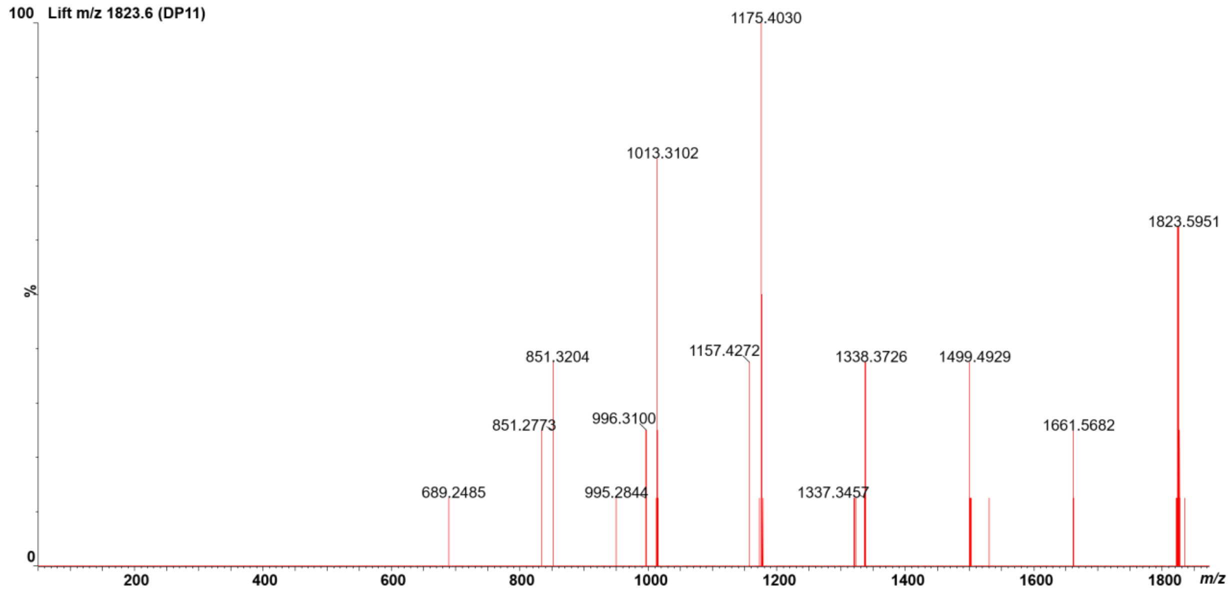

Tandem mass spectrum MS^2 of DP12 ion over a spotted extract of agave stem.

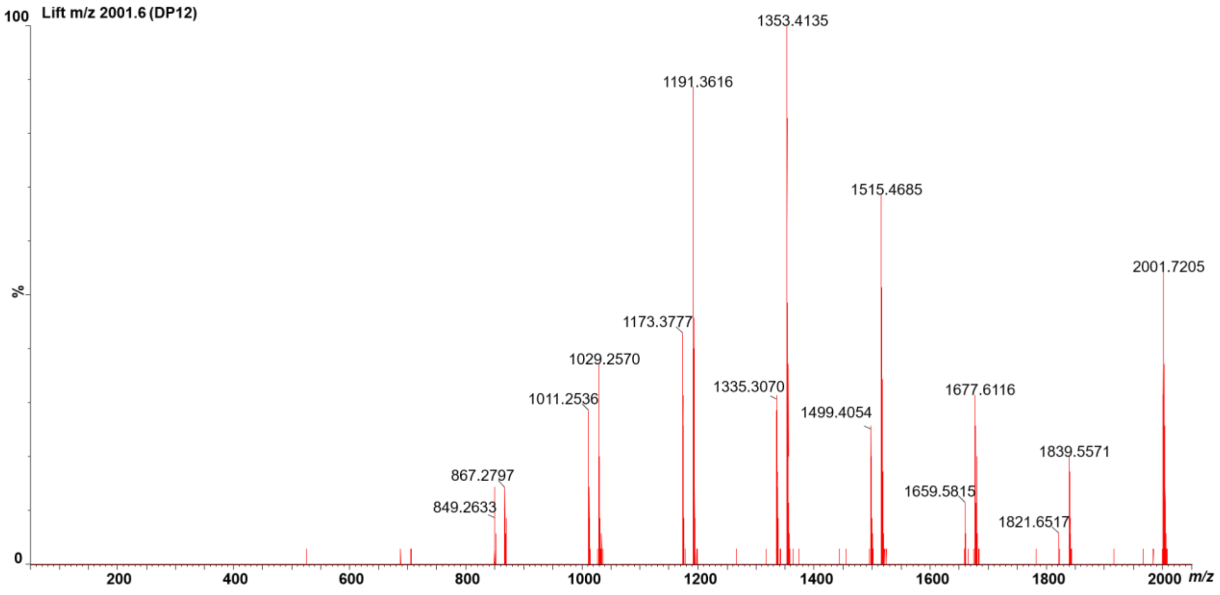

Tandem mass spectrum MS^2 of DP13 ion over a spotted extract of agave stem.

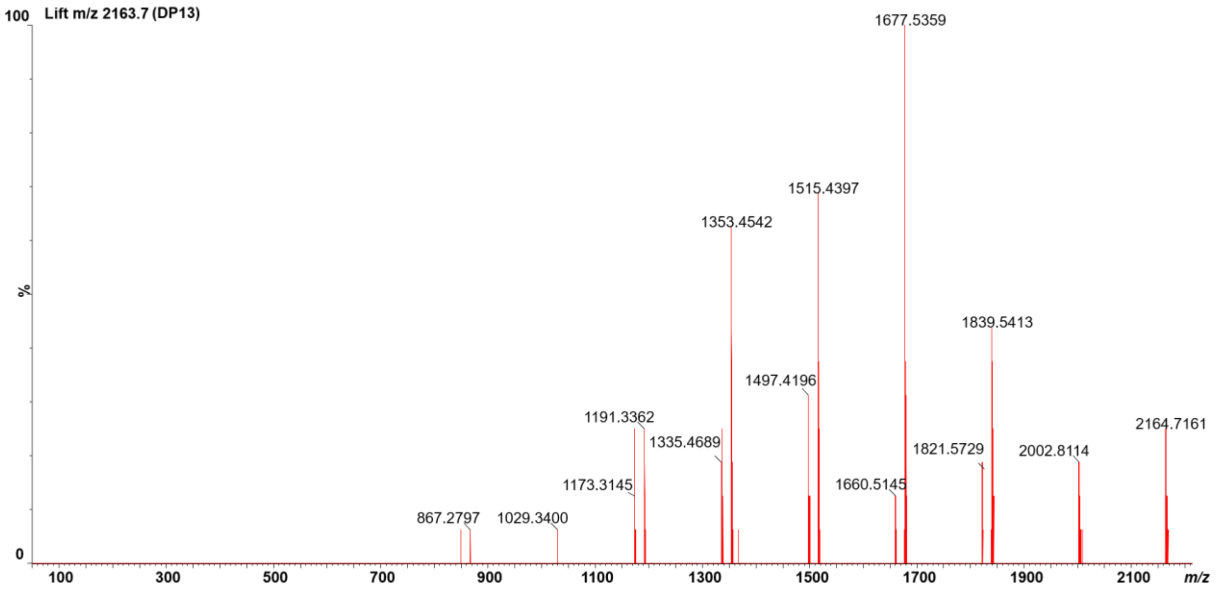

Tandem mass spectrum MS^2 of DP14 ion over a spotted extract of agave stem.

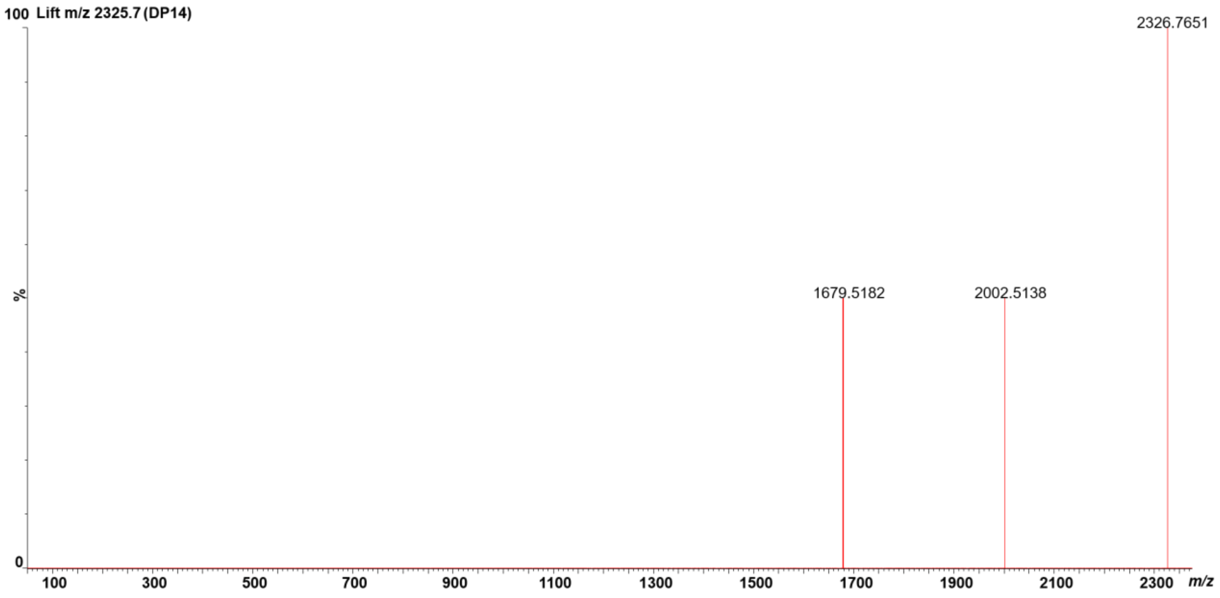

Tandem mass spectrum MS^2 of DP15 ion over a spotted extract of agave stem.

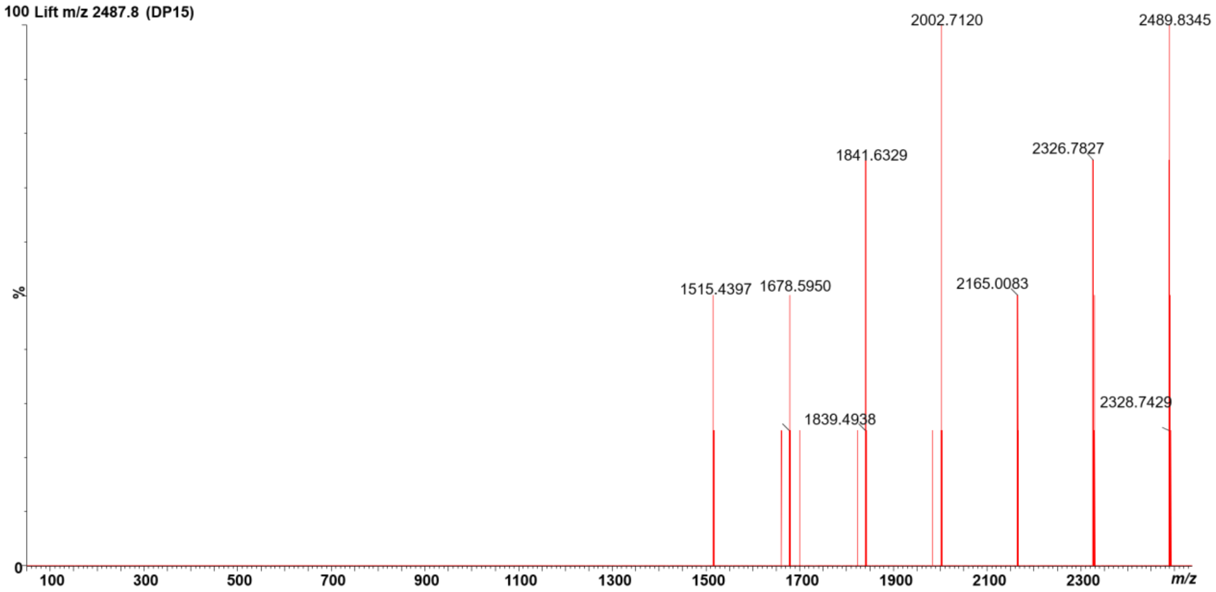

Tandem mass spectrum MS^2 of DP16 ion over a spotted extract of agave stem.

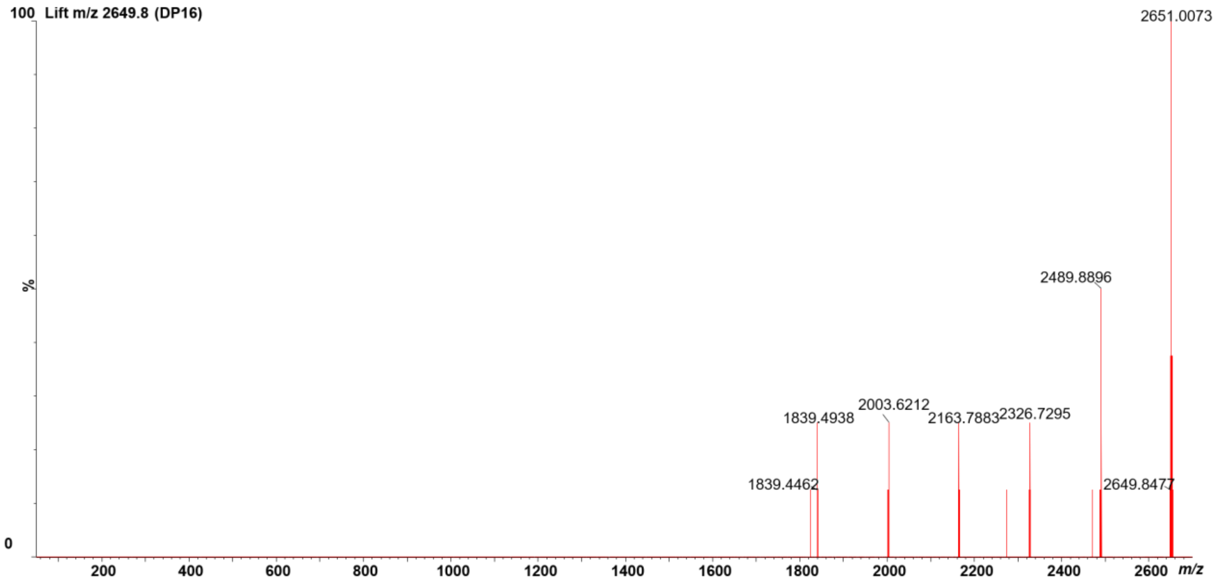

Tandem mass spectrum MS^2 of DP17 ion over a spotted extract of agave stem.

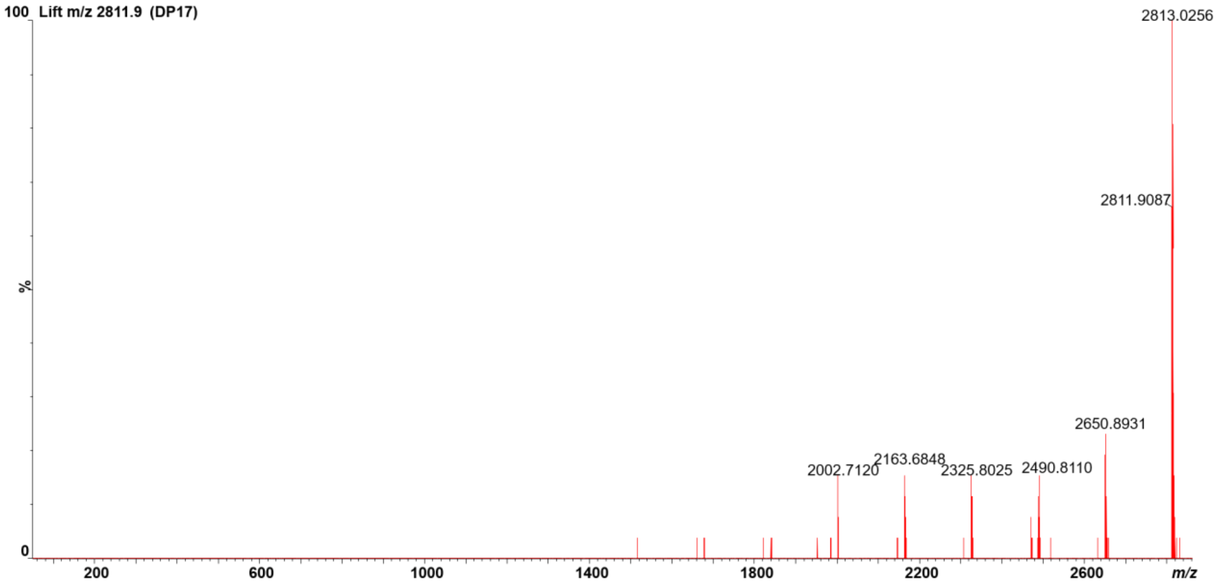

Tandem mass spectrum MS^2 of DP18 ion over a spotted extract of agave stem.

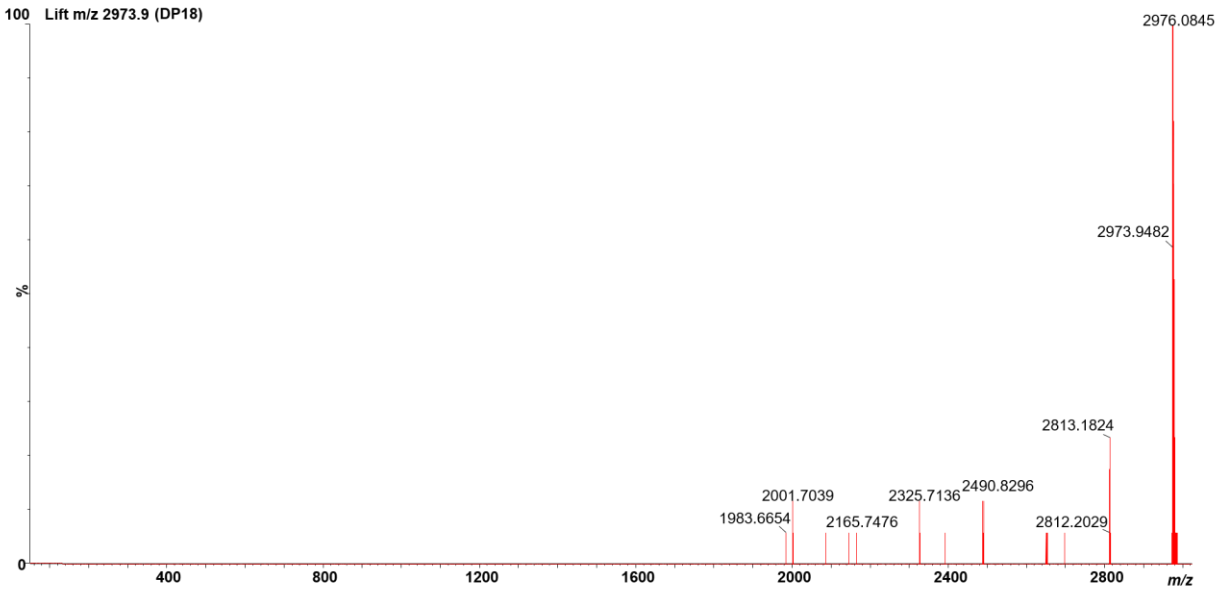

Supplement: Supplementary Figure 7 — MSMS fragmentation data and MSMS spectra of fructans DP3 to DP18. [file Image_7.pdf]
