# Supplementary material for: Localization and Composition of Fructans in Stem and Rhizome of Agave tequilana Weber var. azul
Source: Front Plant Sci. 2021 Jan 20;11:608850. doi: 10.3389/fpls.2020.608850 (PMC7855178; doi:10.3389/fpls.2020.608850)
Supplement: Supplementary Table 1 — Collision cross-sections (Å2) for singly charged protonated oligomers of poly-(D/L)-alanine measured in nitrogen as drift gas. [file Table_1.pdf]

Sequence	(M+H)+	Database[1]	Experimental	Average	StdDev	% RSD
A_5	374.204	181	179.1	180.03	1.37	0.76
A_6	445.241	195	192.2	193.61	1.97	1.02
A_7	516.280	211	206.3	208.65	3.32	1.59
A_8	587.320	228	224.7	226.35	2.34	1.03
A_9	658.350	243	236.3	239.67	4.72	1.97
A_{10}	729.390	256	248.9	252.44	5.03	1.99
A_{11}	800.430	271	260.2	265.58	7.67	2.89
A_{12}	871.460	282	274.0	277.98	5.69	2.05
A_{13}	942.500	294	287.1	290.57	4.85	1.67
A_{14}	1013.540	306.0	300.0	303.01	4.23	1.40

1) From: Busch, M.F., Campuzano, I.D., Robinson, C.V., Anal chem 2012, 84, 7124-7130
